# Chromosome aberrations among atomic-bomb survivors exposed in utero: updated analysis accounting for revised radiation doses and smoking

**DOI:** 10.1007/s00411-021-00960-4

**Published:** 2022-02-17

**Authors:** John Cologne, Hiromi Sugiyama, Kanya Hamasaki, Yoshimi Tatsukawa, Benjamin French, Ritsu Sakata, Munechika Misumi

**Affiliations:** 1 Department of Statistics, Radiation Effects Research Foundation, 5-2 Hijiyama Park, Minami-ku, Hiroshima, 732-0815 Japan; 2 Department of Epidemiology, Radiation Effects Research Foundation, Hiroshima, Japan; 3 Department of Molecular Biosciences, Radiation Effects Research Foundation, Hiroshima, Japan; 4 Department of Clinical Studies, Radiation Effects Research Foundation, Hiroshima, Japan; 5 Department of Biostatistics, Vanderbilt University Medical Center, Nashville, TN USA

**Keywords:** Chromosome translocations, Radiation dose response, In utero radiation exposure, Smoking

## Abstract

A previous study of peripheral blood lymphocyte translocations around age 40 among atomic-bomb survivors exposed in utero revealed no overall association with radiation dose—despite a clear association between translocations and dose among their mothers—but the data suggested an increase at doses below 100 mGy with a definite peak. That analysis of the in utero-exposed survivors did not adjust for their subsequent smoking behavior, an established cause of chromosomal aberrations, or their subsequent exposures to medical irradiation, a potential mediator. In addition, atomic-bomb survivor radiation dose estimates have subsequently been updated and refined. We therefore re-estimated the dose response using the latest DS02R1 dose estimates and adjusting for smoking as well as for city and proximal–distal location at the time of exposure to the atomic bomb. Sex of the survivor, mother’s age around the time of conception, and approximate trimester of gestation at the time of exposure were also considered as explanatory variables and modifiers. Precision of the estimated dose response was slightly lower due to greater variability near zero in the updated dose estimates, but there was little change in evidence of a low-dose increase and still no suggestion of an overall increase across the entire dose range. Adjustment for smoking behavior led to a decline in background number of translocations (the dose–response intercept), but smoking did not interact with dose overall (across the entire dose range). Adjustment for medical irradiation did not alter the association between dose and translocation frequency. Sex, mother’s age, and trimester were not associated with number of translocations, nor did they interact with dose overall. Interactions with dose in the low-dose range could not be evaluated because of numerical instability.

**Supplementary Information:**

The online version contains supplementary material available at 10.1007/s00411-021-00960-4.

## Introduction

Chromosome aberrations—especially translocations—remain a key biological dosimeter for assessing radiation doses long after exposure (McKenna et al. 2019) and a particular appeal is that an actual biological effect (clastogenicity) is measured. For that reason, it was surprising that translocation frequencies (TF) scored in peripheral blood lymphocytes 40 years after exposure among atomic-bomb survivors exposed in utero did not display a dose response over the entire range of doses, even though their mothers clearly showed such a dose response (Ohtaki et al. 2004). This poses a conundrum because it is believed that abdominal radiographic examination of a mother during pregnancy (generally at doses around 10 mGy) is potentially associated with subsequent risk of childhood cancer, including leukemia (Wakeford 1995; Doll and Wakeford 1997; Wakeford and Little 2002). In addition, in utero exposure to doses above 80 mGy has been suggested to be associated with hematologic malignancies later in life in a pooled analysis of cohorts of Southern Urals residents (Schüz et al. 2017), although one study did not reveal an association between in utero radiation exposure and solid cancer or hematologic malignancies in adulthood among Techa River residents (Krestinina et al. 2017). Controversy surrounding the belief that irradiation in utero is a cause of subsequent cancer might in part be due to observational study biases and relatively small study sizes (Hamasaki and Nakamura 2019). The level of risk of childhood cancer has been difficult to estimate precisely, but many authors have concluded that a risk exists at low doses (e.g., Wakeford and Little 2003). Schulze-Rath et al. (2008) argued that the lack of consistency between case–control and cohort study findings should not be taken as evidence of lack of a risk, and Wakeford (2021) recently reviewed the relevant studies and concluded that there is evidence for increased risk of most types of childhood cancer following in utero exposure to low doses of radiation. Because of mechanistic uncertainties, evidence of persistent clastogenic effects of radiation exposure during fetal development could be informative.

Results of animal studies have been consistent with the in utero-exposed atomic-bomb survivor results in that no overall association has been seen between fetal irradiation at relatively high doses and TF in lymphocytes during adulthood (Nakano et al. 2007). Interestingly, though, experiments on in utero-exposed mice revealed elevated TF in mammary and thyroid cells after birth (Nakano et al. 2014; Hamasaki et al. 2016). Studies of chromosome aberrations following fetal radiation exposure in mice typically involved rather high doses (e.g., 2 Gy or higher), whereas the analysis of lymphocyte translocations in the in utero-exposed atomic-bomb survivors included few persons with doses 2 Gy or higher but revealed an increase in TF restricted to doses below 100 mGy with a peak around 30 mGy (Ohtaki et al. 2004). The cause of that low-dose increase is not clear and mechanisms of chromosomal damage repair or elimination of damaged cells prior to establishment of the mature immune system, still poorly understood, are currently under study (Hamasaki and Nakamura 2019).

The previous analysis of TF among in utero-exposed atomic-bomb survivors conducted at the Radiation Effects Research Foundation (RERF) did not account for their smoking behavior, although smoking is associated with the generation of translocations in peripheral blood lymphocytes (Sigurdson et al. 2008), nor did it account for medical radiation exposures, which could be associated with both atomic-bomb radiation dose and generation of translocations (i.e., as a post-exposure mediator). Another important factor is age at the time of TF measurement, but that is not relevant in the study of in-utero exposed atomic-bomb survivors because all participants were studied around age 40 years. Reasons for considering smoking include (1) potential reduction in residual variation leading to greater statistical power and (2) possible adjustment for unmeasured confounding if smoking were related to (acts as a surrogate of) factors that existed prior to—and were associated with—atomic-bomb radiation exposure, if such factors are also associated with the generation of translocations. Such factors might include socio-economic status, as radiation dose strongly depends on ground distance from the hypocenter of the bomb, which—being related to geographic location—is potentially related to occupation, lifestyle, access to medical care, and other factors that could be correlated with exposure to clastogens. Data are not available on such factors per se, but city, proximal–distal exposure location (based on cutoff at 3 km), and their interaction (which is related to urban–rural differences near the atomic-bomb hypocenters of the two cities) have recently been used as proxies for potential but unmeasured confounders (French et al, 2018). In addition, since the time of the analysis by Ohtaki et al. (2004), atomic-bomb survivor radiation dosimetry has been updated from the former DS86 system (Roesch 1987) to the DS02 system (Young and Kerr 2005; Cullings et al. 2006), and the dose estimates (now labeled DS02R1) were recently improved by increasing the accuracy of estimated location at the time of exposure and allowing for shielding by features of nearby terrain (Cullings et al. 2017).

The purpose of the present analysis is therefore threefold. First is to assess whether the results differ with the new DS02R1 atomic-bomb survivor dose estimates. Second is to adjust for two additional factors: smoking behavior at the time of blood collection for translocation scoring and estimated medical radiation exposures known to have occurred prior to translocation scoring. Third is to evaluate effects on TF of other variables (sex, mother’s age at conception, and trimester at the time of exposure) and to investigate possible interactions between these variables and radiation dose. Interactions are limited to the overall slope across the entire dose range; it was not possible to fit interactions in the low-dose region, for reasons that will become apparent below. The analyses reported here are more thorough than those previously reported by Ohtaki et al. (2004), in particular through the addition of diagnostics and alternative approaches.

## Methods

### Study participants and data

A cohort of atomic-bomb survivors who were exposed in utero is being followed by the Radiation Effects Research Foundation (Sugiyama et al. 2021). The Adult Health Study (AHS) is a clinical program established in 1958, comprising atomic-bomb survivors who were exposed to the atomic bombings in Hiroshima or Nagasaki. In 1978, some individuals exposed in utero (*n* = 1021) were added to the AHS cohort and followed through biennial health examinations. The AHS in utero cohort, formed in 1959, included a group of individuals exposed in utero within 2000 m of the hypocenters at the time of the bombings who lived in Hiroshima or Nagasaki as of October 1, 1950 (proximally exposed) and a comparison group—matched by city, sex, and month of birth—randomly selected from among in utero-exposed survivors who were distally located at the time of the bombing (3000–4999 m; distally exposed). Dose to in utero-exposed survivors is estimated using mother’s uterus dose from the DS02 dosimetry system with values as recently revised (DS02R1; Cullings et al. 2017). Because no participants were selected who were exposed between 2 and 3 km, there is a gap between 2.05 and 19.96 mGy in the dose estimates; no distally exposed participants had doses above 1.53 mGy, and only three proximally exposed participants had doses below 19.96 mGy (all three were exposed to 2.05 mGy or less). Estimates of trimester at the time of exposure, which are back-calculated from date of birth assuming (perhaps incorrectly) full-term pregnancy, were obtained from an internal database and differ slightly from those used by Ohtaki et al. (2004) as a result of subsequent institutional reassessment of dates of birth.

Blood samples for translocation scoring were obtained from a sample of AHS in utero cohort participants during two consecutive biennial exam cycles beginning in 1985, when the in utero-exposed survivors were about 40 years of age. Participants at the biennial exams were interviewed by nurses to assess smoking behavior. Smoking behavior was categorized as current-, former-, or non-smoker on the basis of the two questionnaires closest to the time of blood drawing. If the two questionnaires during the study period contained conflicting answers, we assigned for purposes of analysis the highest smoking behavior in the order current > former > never (such inconsistencies were rare). No data were available on passive smoke exposure. Medical radiation exposures were assessed from two sources: cumulative diagnostic X-ray doses to bone marrow and gonads through 1982 were estimated by Yamamoto et al. (1988), and self-reported exposures to radiation therapy were assessed through review of the biennial clinical examination records.

Ohtaki et al. (2004) performed translocation scoring with G-banding, which reveals the total number of translocations in each of *N* cells scored (typically, but not always, 100 cells). The frequencies so obtained are, strictly speaking, Poisson-distributed counts. However, multiple translocations per cell were unlikely given the small mean frequency in the in utero study, and there were no clonal aberrations. We therefore considered the total TF in each participant as a binomially distributed variable with denominator *N* and mean *p*_*θ*_ (the probability that a cell has one or more translocations) depending on parameters in a vector *θ*, as was done by Ohtaki et al. (2004); a simulation comparing the binomial distribution with *N* trials and probability 0.01 to a Poisson distribution with expected value 1 (i.e., mean count of one aberration per cell) revealed that these two distributions are nearly identical (not shown). Elements of the vector *θ* are defined in detail below. Because the number of cells successfully scored for translocations varied among participants, the TF scores are not directly comparable across participants. We therefore based analyses on the translocation proportion (frequency of translocations per 100 cells: TF/*N*), which we express as a percentage: TF% = 100 × TF/*N*.

Ohtaki et al. (2004) investigated the association between TF% and radiation dose using mothers’ uterus dose estimates from the DS86 dosimetry system. In the current analysis we used DS02R1 mothers’ uterus dose estimates. In both cases, the dose estimate is weighted absorbed dose in mGy with an RBE weight of 10 for neutron relative to gamma dose. Ohtaki et al. (2004) initially assessed the association between TF% and radiation dose with nonparametric smoothing, and we reproduce that analysis with the DS02R1 dose estimates. Global smoothers (such as lowess, Fox and Weisberg 2018) are appropriate for assessing overall association, but because of the skewed dose distribution a locally adapted bandwidth smoother (super smoother, Friedman 1984; implemented as R function **supsmu**) was applied. The fraction of observations included in local fits of the running lines smoother (the span) was determined by cross-validation, and various degrees of smoothness were imposed (the bass argument to the **supsmu** function, with 1 being the least smooth and 10 being the smoothest).

### Dose–response model and methods of statistical analysis

On the basis of the pattern revealed by nonparametric smoothing and in consideration of the seemingly contradictory findings—that no overall association between fetal exposure and lymphocyte TF was observed among atomic-bomb survivors or in animals exposed in utero despite evidence of childhood and adult-onset leukemia in studies of in utero exposure to low doses—Ohtaki et al. (2004) postulated a four-parameter nonlinear dose–response model that included, in addition to an intercept parameter, three radiation dose parameters: an initial slope multiplied by an exponential downturn and a separate overall linear slope across the entire dose range. This dose–response model for *p*_*θ*_, the mean translocation proportion, is

1 $$p_{\theta } = \theta_{1} + \theta_{2} de^{{ - \theta_{3} d}} + \theta_{4} d,$$

where *d* is mother’s weighted uterus radiation dose in mGy. We refer to this model as “the basic four-parameter dose–response model” (or simply “the basic dose–response model”). Although the scale of the outcome is a proportion (between 0 and 1), we show results as percentages. We show results for the initial slope parameter (*θ*_2_) in units of percent per mGy and for the overall slope parameter (*θ*_4_) in units of percent per Gy.

To the model (1) we can add terms involving variables that affect the intercept, such as city and proximal–distal location at the time of exposure (and their interaction to capture urban–rural differences by city), smoking category, sex, mother’s age around the time of conception, and trimester of gestation at the time of exposure. Because the precise date of conception is unknown, we use mother’s age at the time of the bombing as a surrogate, since all conceptions of in utero-exposed survivors must have occurred less than one year before the bombing. Trimester at the time of bombing was considered because it is potentially associated with dramatic changes in the environment and lifestyle directly associated with the bombing and because radiation exposure can be detrimental in early gestation (Gök et al. 2015). Terms can also be added for variables that might interact with radiation by including cross-products of dose with those variables. Cumulative dose of medical radiation exposure can in principle be thought of as a potential mediator, since more frequent diagnostic procedures and radiation therapy could be associated with prior atomic-bomb radiation exposure; we simply incorporated cumulative medical radiation dose as if it were a confounder, using either a simple linear term or the same form of dose–response model as was used for atomic-bomb dose. Extended models are defined in the Results section when needed. Models were fit with constrained maximum likelihood using the **maxLik** package in R (Henningsen and Toomet 2011) with non-negativity constraints applied to the overall intercept parameter and all dose-related parameters.

Constrained maximum likelihood can invalidate standard asymptotic inference based on estimated standard errors (such as Wald tests and confidence intervals), so we computed 95% confidence regions based on bootstrapping (Davison and Hinkley 1997) for the individual parameters and for the fitted curve. Nonparametric bootstrapping (sampling with replacement from the empirical distribution of the observations) was used to generate 1,000 samples with the **boot** command in the R **boot** package. The constrained maximum likelihood procedure was applied to each bootstrap sample to obtain bootstrap estimates of the median and 95% confidence bounds for each estimated parameter. Bootstrap confidence bands for the fitted dose–response curve were obtained by bootstrapping the entire fitted curve and taking pointwise 2.5th and 97.5th percentiles of the fitted values at each of a large number of pre-specified dose values. The pre-specified dose values were generated as uniform between 0 and 72 on the log dose scale, resulting in a skewed dose distribution between 1 and 1339 on the mGy scale and were supplemented with ten equally spaced values between 0 and 0.9 mGy. To assess influence of individual observations we applied the jackknife-after-bootstrap method (Efron 1992) implemented as the **jack.after.boot** function in the R **boot** package (see the Online Resource, Sect. 3.2, for details). To assess overdispersion, we computed the dispersion factor estimate based on weighted residual sum of squares that is recommended by McCullagh and Nelder (1989, Eq. 4.23). As a check on the estimated model parameters and confidence regions, we also fit the basic dose–response model with the empirical Bayes approach (Carlin and Louis 2009) as implemented in the OpenBUGS software (Lunn et al. 2013) called from R with the **BRugs** package (version 0.9–0, Thomas et al. 2006). As with the constrained maximum likelihood fitting, the likelihood was assumed to be binomial with denominator *N*. See the Online Resource (Sect. 4) for further details.

After linking the updated data to the original translocation data, we replaced the institutional ID numbers with randomly generated ID numbers for purposes of computing and outputting results. Analyses were performed with R version 3.6.3 running under Windows 10 on a 64-bit Toshiba Dynabook computer. OpenBUGS version 3.2.3 rev 1012 (2014-03-15) was used for the empirical Bayes approach.

## Results

Characteristics of the study cohort are described in Table 1. These are the same individuals as were described by Ohtaki et al. (2004) with the exception of one whose mother of record was subsequently determined not to be the birth mother, so that person’s dose is unknown. The “distally exposed” individuals generally have dose estimates of zero. Persons who were exposed proximally (less than 2 km from the hypocenter of the bomb) were near the city center in Hiroshima, whereas the hypocenter of the bomb in Nagasaki was in a more industrial-rural area.

**Table 1:** Participant characteristics on factors used in the analysis

City	Proximally exposed (< 2 km)	Distally exposed (≥ 3 km)	Total
Hiroshima	125	138	263
Nagasaki	26	41	67
Total	151	179	330

Sex	Non-smoker	Current smoker	Former smoker	Total
Males	33	117	31	181
Females	129	17	3	149
Total	162	134	34	330

Mother’s DS02R1 uterus dose (mGy)	First trimester	Second trimester	Third trimester	Total
< 5	58	75	49	182
[5, 50)	12	9	7	28
[50, 100)	12	8	9	29
[100, 200)	9	16	14	39
[200, 500)	7	16	11	34
500 + [maximum (mGy)]	3 [1091.8]	12 [1306.8]	3 [1008.6]	18
Total	101	136	93	330

**Fig. 1:**
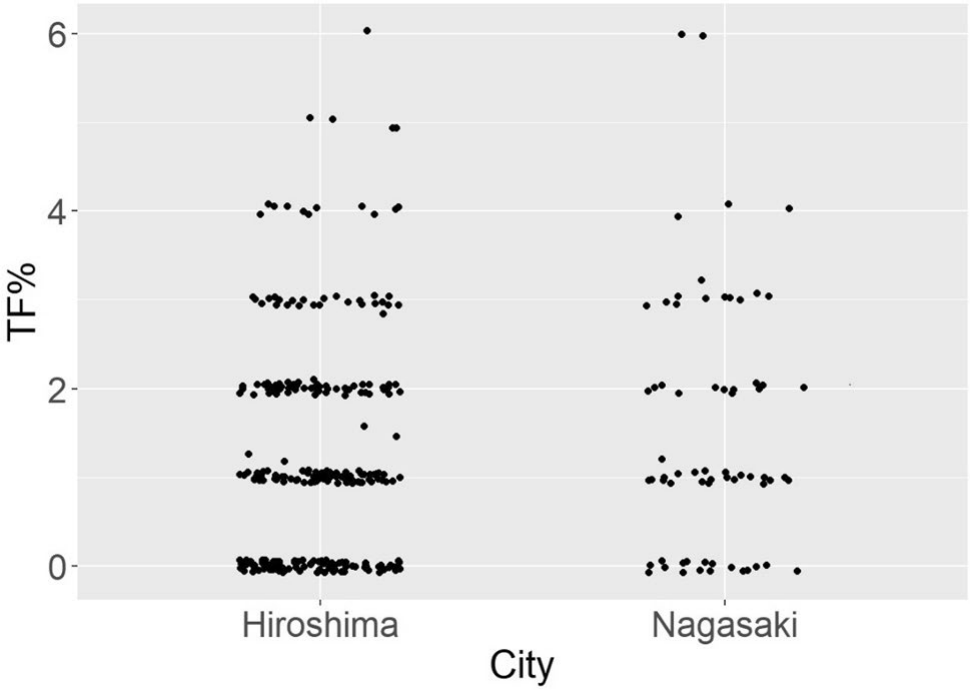
TF% (100 × translocation frequency divided by number of cells scored) by city in which exposed

Distributions of TF% by city are shown in Fig. 1 (slightly jittered along both axes to render the points distinguishable). Most of the values are based on a total of 100 cells scored. In 31 of the samples fewer than 100 cells were scored, with number of scored cells in those samples ranging from 58 to 99 (16 samples had more than 90 but less than 100 cells scored). Thus, values of TF% are usually the same as, and otherwise generally close to, values of TF, so that most of the points on Fig. 1 are exact multiples of 1%.

**Fig. 2:**
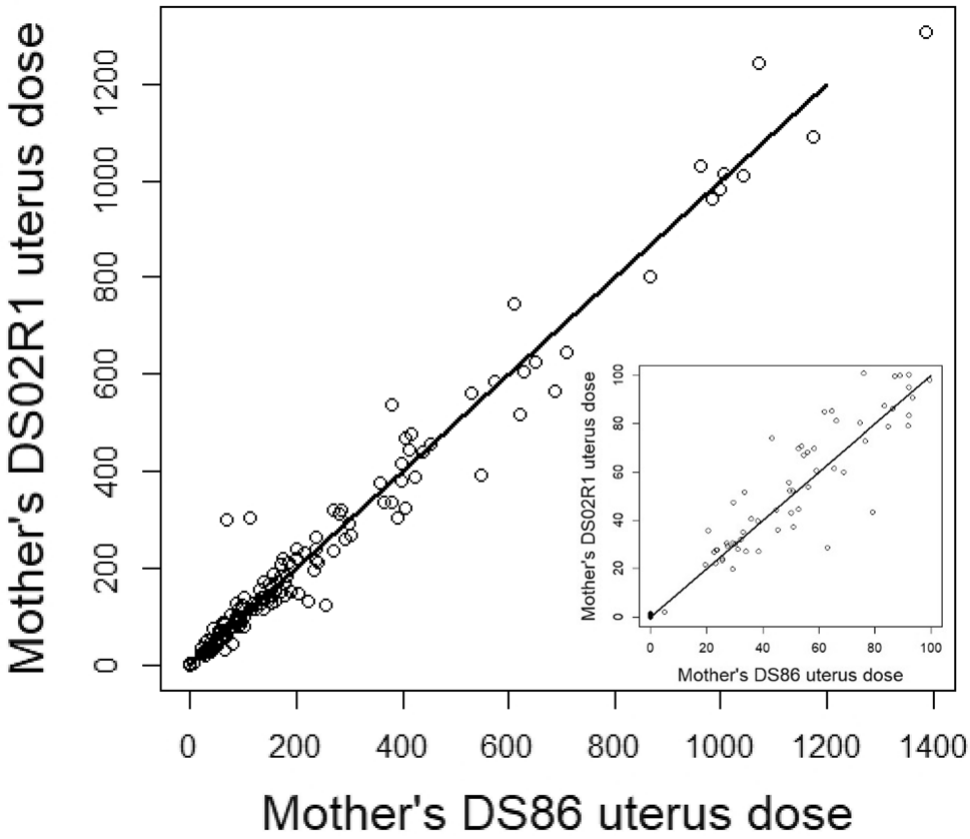
Bivariate scatterplot of DS86 and DS02R1 mothers’ uterus dose estimates. The inset shows the region of the plot below 100 mGy

Figure 2 shows generally good agreement between DS02R1 and DS86 mothers’ uterus dose estimates. The primary difference is that, whereas estimated doses below 5 mGy were assigned value 0 under the DS86 system because it was thought that the system did not justify such small precision, the DS02 system resulted in many of these having non-zero estimated values (albeit these are quite small—the cluster of points at DS86 value zero in the inset to Fig. 2). Nevertheless, as noted above, there is a gap in the low-dose region, with no DS02R1 doses between about 2 and 20 mGy. Potential influence of the two outlying points with DS86 < 200 mGy but DS02R1 > 200 mGy was assessed but found to be unimportant (data not shown).

Nonparametric smooths of the data with DS02R1 fit with super smoother and the maximum degree of smoothing (bass 10) are shown in the Online Resource (Sect. 2, Fig. S2). As with the smoothing analysis based on DS86 shown in the report by Ohtaki et al. (2004), there is an increase followed by a downturn to near baseline level in a restricted region of the low-dose range. Not only is this pattern evident with the full data, it is also seen in each of the mutually exclusive partitions defined by estimated trimester at the time of exposure. Because the super smoother is based on local adaptive fitting, the increase and downturn are estimated on the basis of observations below the gap between 2 and 20 mGy. Therefore, we do not believe that these smooths necessarily reveal the true range of the low-dose effect, since there are no data in the 2–20 mGy region.

**Fig. 3:**
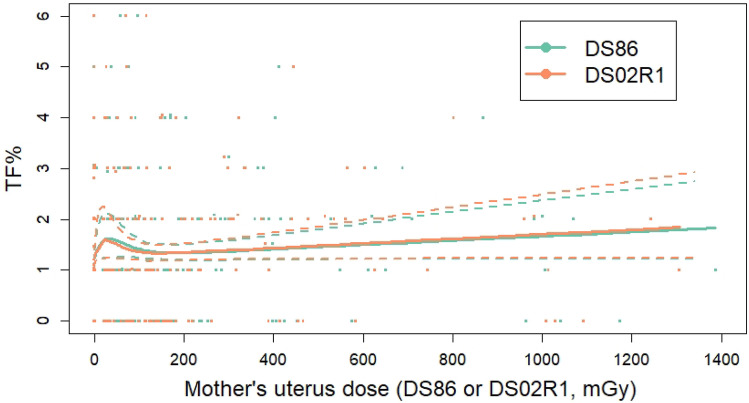
Fits of the basic four-parameter dose–response model to the translocation frequency data with DS86 or DS02R1 dose estimates

Table 2 presents parameter estimates for the basic dose–response model (1) fitted with constrained maximum likelihood applied to mothers’ DS86 and DS02R1 uterus doses, along with results of bootstrapping. All four parameters were constrained to be non-negative. Figure 3 shows the corresponding fitted dose responses. The fitted parameters based on DS86 are slightly different from those reported by Ohtaki et al. (2004) because a different numerical program was used (Gauss **CML** was used by Ohtaki et al., whereas R **maxLik** was used in the present analyses). With DS02R1 dose estimates the peak is at a slightly lower dose and the confidence bands are slightly wider than those obtained with DS86, but the change in dose estimates had no noteworthy effect on the fitted dose response.

**Table 2:** Constrained maximum likelihood parameter estimates for the basic four-parameter dose–response model

Parameter	Estimate	Bootstrap median	Bootstrap lower bound (2.5th percentile)	Bootstrap upper bound (97.5th percentile)
DS86 (maximum log-likelihood = − 502.60)^a^				
*θ*_1_—intercept (%)	1.25	1.24	1.08	1.41
*θ*_2_—initial slope (% / mGy)	0.031	0.034	0.0	0.107
*θ*_3_—downturn (mGy^−1^)	0.032	0.032	0.028	0.049
*θ*_4_—overall slope (% / Gy)	0.421	0.436	0.0	1.26
DS02R1 (maximum log-likelihood = − 503.18)				
*θ*_1_—intercept (%)	1.26	1.25	1.08	1.41
*θ*_2_—initial slope (% / mGy)	0.037	0.035	0.0	0.156
*θ*_3_—downturn (mGy^−1^)	0.042	0.042	0.032	0.057
*θ*_4_—overall slope (% / Gy)	0.451	0.456	0.0	1.33

The model is that of Eq. (1)

^a^Corresponding estimates (95% CI) of the four parameters reported by Ohtaki et al. (2004) were 1.2 (1.1, 1.4) %, 0.038 (0, 0.103) %/mSv, 0.0335 (0.007, 0.0655) /mSv, and 0.5 (0, 1.4) %/Sv, respectively

The profile likelihood for the two low-dose parameters (*θ*_2_ and *θ*_3_) in the basic dose–response model with DS02R1 dose estimates is shown in the Online Resource (Sect. 3.3, Fig. S6). Although the profile likelihood surface for the two low-dose parameters is poorly behaved near the origin due to non-identifiability of the downturn parameter when the initial slope is zero, the surface has concentric contours in the region around the constrained maximum likelihood estimate, signifying that the data are consistent with non-zero values of these two parameters. The non-identifiability is therefore not expected to have a strong effect on the model fit as long as reasonable starting values are used (using the origin—{0,0}— as starting values, as is typically done in parameter estimation, would be meaningless in the case of these two parameters). If the data were not consistent with a low-dose effect of radiation, the constrained maximum likelihood iterations should tend toward the origin and fail to converge. However, this does not mean that the constrained maximum likelihood estimates are precise; we observed some instability in estimating the two low-dose parameters, which we attribute to their interdependence, the small amount of low-dose data, and the gap in observations between 2 and 20 mGy.

The fit of the basic four-parameter dose–response model was confirmed with the alternative, empirical Bayes, approach. Details are provided in the Online Resource (Sect. 4).

Before proceeding to add other explanatory variables to the basic four-parameter dose–response model, we estimated a joint model in atomic-bomb dose and cumulative medical diagnostic X-ray dose by fitting analogous parameters to both dose estimates among all but 22 individuals whose X-ray dose was unknown. Only diagnostic X-ray dose estimates were used for the medical exposures because review of biennial clinical examination records revealed only one reported occasion of radiation therapy among the study participants: an individual who had a translocation proportion of 2% and a maternal DS02R1 uterus dose of 0 mGy. Nonparametric smooth fits of TF% to X-ray dose did not reveal an increase with dose (not shown) and adjusting the basic four-parameter dose response model for X-ray dose did not substantially affect the parameter estimates for the effect of atomic-bomb dose (results shown in the Online Resource, Sect. 5, Tables S6 and S7).

Maximum likelihood estimates of parameters for predictors of TF%, apart from smoking and sex, are shown in Table 3 along with bootstrap confidence interval estimates. Any difference in overall TF% by sex could be confounded by smoking, so sex will be examined later, after smoking has been added to the model. As mentioned previously, city, distance, and their interaction were included to account for possible confounding. The maximum mother’s age at the time of the bombing was 44 and few mothers were aged 40 or older at the time of the bombing (the distribution is depicted in the Online Resource, Sect. 1, Fig. S1). None of these variables contributed to better model fit, so only the city-distance terms were retained (because of concern over possible confounding) when incorporating the other factors. Surprisingly, adding further covariates to the model did not necessarily lead to an increase in log-likelihood; we attribute this to instability in estimating the two low-dose parameters.

**Table 3:** Parameter estimates (95% bootstrap confidence intervals) with additional factors added to the intercept of the nonlinear model

Parameter	Terms added to the basic four-parameter dose–response model		
	City and distance Loglik^a^ = − 500.91	Mother’s age Loglik = − 501.21	Trimester Loglik = − 500.81
*θ*_1_—intercept (%)	1.50 (0.94, 1.74)	1.33 (0.92, 1.71)	1.53 (0.94, 1.90)
*θ*_2_—initial slope (%/mGy)	0.016 (0.0, 0.11)	0.027 (0.0, 0.136)	0.015 (0.0, 0.13)
*θ*_3_—downturn (mGy^−1^)	0.048 (0.027, 0.052)	0.038 (0.025, 0.061)	0.042 (0.032, 0.054)
*θ*_4_—overall slope (%/Gy)	0.071 (0.0, 1.5)	0.32 (0.0, 1.55)	0.17 (0.0, 1.6)
*θ*_5_—city (Δ%^b^) (coded as ± 1)	0.094 (− 0.21, 0.40)	0.024 (− 0.18, 0.38)	0.13 (− 0.17, 0.36)
*θ*_6_—distal (Δ%)	− 0.26 (− 0.48, 0.29)	− 0.15 (− 0.42, 0.36)	− 0.14 (− 0.52, 0.33)
*θ*_7_—Nagasaki–distal interaction (Δ%)	0.17 (− 0.67, 0.91)	0.29 (− 0.55, 0.74)	0.024 (− 0.55, 0.80)
*θ*_8_—mother’s age (Δ%/5 years)	–	0.022 (− 0.092, 0.13)	–
*θ*_9_—1st trimester (Δ%)	–	–	− 0.10 (− 0.52, 0.30)
*θ*_10_—2nd trimester (Δ%)	–	–	− 0.11 (− 0.49, 0.21)
Reference—3rd trimester	–	–	0

The model is $p_{\theta } = \theta_{1} + \left[ {\theta_{2} de^{{ - \theta_{3} d}} + \theta_{4} d} \right] + \theta_{5} c + \theta_{6} g + \theta_{7} \left( {n \times g} \right) + \theta_{8} m + \theta_{9} t_{1} + \theta_{10} t_{2}$, where *d* is mother’s uterus radiation dose, *c* is city (coded ± 1), *g* is distal ground distance (≥ 3 km), *m* is mother’s age at exposure (centered at the mean, 29.4 years), and *t*_1_ and *t*_2_ are indicators of first and second trimester, respectively (note that not all parameters were fit simultaneously)

^a^Log-likelihood

^b^Δ% is difference from overall mean translocation percentage; Hiroshima coded − 1, Nagasaki coded + 1

At this point we performed two sensitivity analyses. The seven-parameter model of Table 3 (basic dose response with the addition of the city-distance terms; parameters 1 through 7 in the first column of estimates in Table 3) was chosen for these analyses because it reflects our best estimate of the dose response with adjustment for potential confounders. First, as a check of sensitivity of the fitted parameter estimates to the non-negativity constraints, we applied the maximum likelihood method without the constraints on the three dose-related parameters. The results (detailed in the Online Resource, Sect. 3.1, Table S3) did not show any important difference between unconstrained and constrained fits, but the constraint on the overall intercept was required to achieve stable fits because, without it, the binomial mean proportion can be negative, which causes an error when the optimization routine calls the likelihood function. Second, we applied the jackknife-after-bootstrap diagnostic procedure to assess influence of individual observations. The results (detailed in the Online Resource, Sect. 3.2, Fig. S3) did not reveal any noteworthy influence. The estimated value of the dispersion factor with this model was 1.203. Because we deemed this to be inconsequential and unlikely to be a cause of the shape of the low-dose response, we did not make adjustment for overdispersion.

Distributions of dose and TF% by smoking category are presented in the Online Resource (Sect. 1); from these we can surmise that smoking is not likely to confound the effect of radiation on TF%. Estimates of parameters for the two smoking categories (not including the reference, non-smoker, category) and for sex added to the intercept of the seven-parameter model of Table 3 are shown in Table 4. Also shown in Table 4 are estimates of interactions between the overall radiation dose–response slope and smoking, sex, or trimester of exposure. A main effect of trimester was not included when the trimester–radiation interaction was assessed, and the third trimester was used as the reference group because radiation effects during in utero exposure are most pronounced during the early stages of gestation (Gök et al. 2015). To reduce clutter, estimates of the three city-distance parameters are not shown as these are not the focus of the analysis. The best fit in terms of maximized log-likelihood was for the model with main effects of smoking and sex (log-likelihood − 499.57; AIC 1019.14). However, the fit of this model was not better than that of the model without smoking or sex (log-likelihood − 500.91, Table 3; AIC 1015.82) and the confidence interval for the sex parameter included zero.

**Table 4:** Parameter estimates (95% confidence intervals) for smoking and sex in the intercept as well as interactions with the overall slope for radiation

Parameter		Smoking		Sex		Trimester
		No interaction (− 502.98)	With interaction (− 501.08)	No interaction (− 499.57)	With interaction (− 500.84)	With interaction (− 500.09)
*θ*_1_—Intercept (%)		0.96 (0.77, 1.62)	1.27 (0.82, 1.51)	1.47 (0.93, 1.70)	1.14 (0.84, 1.58)	1.21 (0.91, 1.53)
*θ*_2_—Initial slope (%/mGy)		0.079 (0.002, 0.13)	0.041 (0.0, 0.080)	0.011 (0.0, 0.12)	0.064 (0.0, 0.13)	0.026 (0.0, 0.11)
*θ*_3_—Downturn (mGy^−1^)		0.040 (0.033, 0.056)	0.031 (0.024, 0.036)	0.046 (0.035, 0.047)	0.041 (0.035, 0.050)	0.044 (0.034, 0.050)
*θ*_4_—Overall slope (%/Gy) (with interactions, overall slope for non-smoker, average of sex, or 3^rd^ trimester)		1.1 (0.00005, 1.9)	0.43 (0.0, 2.0)	0.10 (0.0, 1.5)	0.90 (0.00008, 1.85)	0.000005 (0.0, 1.25)
*θ*_5_—city (Δ%) (coded as ± 1)		0.10 (− 0.15, 0.47)	0.12 (− 0.15, 0.36)	0.13 (− 0.11, 0.40)	0.11 (− 0.12, 0.41)	0.04 (− 0.17, 0.39)
*θ*_6_—distal (Δ%)		0.24 (− 0.17, 0.41)	0.08 (− 0.23, 0.35)	− 0.25 (− 0.47, 0.28)	0.14 (− 0.28, 0.39)	− 0.14 (− 0.41, 0.30)
*θ*_7_—Nagasaki–distal interaction (Δ%)		0.17 (− 0.76, 0.64)	0.07 (− 0.46, 0.72)	0.13 (− 0.49, 0.63)	0.09 (− 0.54, 0.76)	0.26 (− 0.48, 0.86)
Smoking (Δ%) (non-smoker is the reference)	*θ*_11_—current *θ*_12_—former	0.15 (− 0.15, 0.47) 0.05 (− 0.42, 0.51)	− 0.086 (− 0.16, 0.54) − 0.19 (− 0.53, 0.31)	0.13 (− 0.19, 0.45) − 0.02 (− 0.22, 0.33)	0.029 (− 0.22, 0.43) − 0.069 (− 0.43, 0.43)	0.27 (− 0.060, 0.47) 0.14 (− 0.34, 0.58)
*θ*_13_—Sex (Δ%; sex coded as − 1 for males, + 1 for females)		–		− 0.052 (− 0.22 0.12)	− 0.14 (− 0.30 0.096)	–
Smoking–radiation interaction (Δ%/Gy) (non-smoker is the reference)	*θ*_14_—current *θ*_15_—former	–	− 0.18 (− 2.4, 0.99) 2.3 (− 1.3, 4.6)	–		–
*θ*_16_—Sex–radiation interaction (Δ%/Gy; sex coded as − 1 for males, + 1 for females)		–		–	0.42 (− 0.46 1.2)	–
Trimester–radiation interaction (Δ%/Gy)		–		–		0.000001 (0.0, 2.18)
*θ*_17_—1st trimester (3rd trimester is the reference)						0.70 (0.0, 1.56)
*θ*_18_—2nd trimester						

^a^The model is $p_{\theta } = \theta_{1} + \left[ {\theta_{2} d{\text{e}}^{{ - \theta_{3} d}} + \theta_{4} d} \right] + \theta_{5} c + \theta_{6} g + \theta_{7} (n \times g) + \theta_{11} s_{{\text{C}}} + \theta_{12} s_{{\text{F}}} + \theta_{13} x + \theta_{14} (s_{{\text{C}}} \times d) + \theta_{15} (s_{{\text{F}}} \times d) + \theta_{16} (x \times d) + \theta_{17} (t_{1} \times d) + \theta_{18} (t_{2} \times d)$, where in addition to variables defined in the model for Table 3, *s*_C_ indicates current smoker, *s*_F_ indicates former smoker, and *x* is sex (males coded − 1, females coded + 1) (note that not all parameters were fit simultaneously)

Although zero is included within the confidence intervals for the smoking parameters related to the intercept, the parameter estimates are consistent with an effect of smoking and the overall intercept estimate is lower after adjustment for smoking. The present study is not appropriate for testing a smoking effect per se, given the relatively small sample size and the fact that smoking has already been shown to be positively associated with chromosome translocation frequencies (Sigurdson et al. 2008). It is noteworthy that, after adjustment was made for smoking and the overall intercept became smaller, the estimates of the initial slope and overall slope both increased in magnitude. With adjustment for smoking there was no qualitative change in the fit of the dose response in the low-dose region, although the lower confidence bound for the initial slope was slightly greater than zero. The lower confidence bound for the overall slope across the entire dose range remained essentially zero. Because the bootstrap is based on random re-sampling, the values 0.002 and 0.00005 for the lower confidence bounds of these two slope parameters might merely be reflections of sampling variability. A plot of profile likelihood contours for the two smoking parameters (added to the 7-parameter basic dose–response model with city-distance adjustment) is shown in the Online Resource (Sect. 3.3, Fig. S7). The profile likelihood surface for the two smoking parameters has concentric ovals, signifying that there should be little difficulty estimating the parameters by maximum likelihood (the smoking parameter fits were not constrained).

With the addition of an interaction between smoking and the overall slope for radiation, the overall slope was reduced and the smoking main effect parameters became negative, while the interaction parameters were widely disparate. This is most likely attributable to random noise, as the confidence intervals for all of these parameters were quite wide. With the addition of an interaction between trimester and the overall slope for radiation, there was large variation among the estimates, with the overall slope being essentially zero for the first and third trimesters, but in the second trimester it was close to the level of overall slope estimated in models without that interaction. Nevertheless, the lower confidence bound for the interaction of overall slope with the second trimester was zero. Examination of the raw data (Fig. 3) reveals that there are few observations in the high-dose range, so categorization by trimester could result in extreme uncertainty. We therefore cannot ascribe any meaning to the relatively large estimate in overall dose–response slope with exposure in the second trimester. Furthermore, the log-likelihood did not suggest an improved fit with the addition of the interaction, so there is no statistical evidence of heterogeneity, by trimester, in the overall slope.

**Fig. 4:**
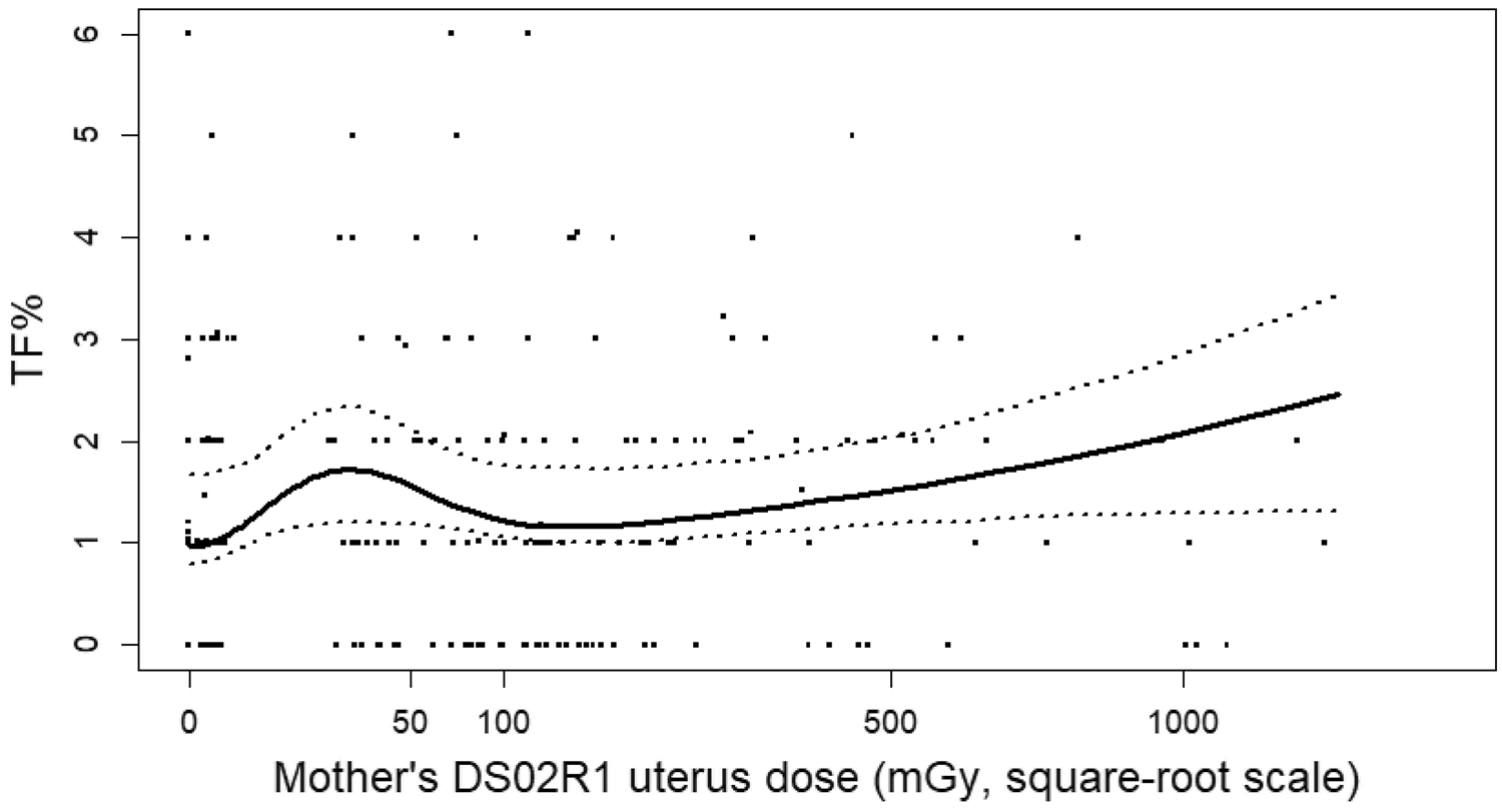
Plot of final dose–response model based on DS02R1 mothers’ uterus dose estimates. The abscissa is on the square root scale, but the fitted curve was obtained using untransformed doses. The fitted line is for proximally exposed non-smokers, equally averaged over city

Based on the model fits described above, we decided on a final model that included, in addition to the basic four-parameter dose–response model, the city-distance parameters (city, distal, and Nagasaki–distal interaction) and the two smoking behavior parameters (first column of Table 4). Figure 4 illustrates this final fitted dose–response model for proximally exposed in utero survivors who were non-smokers, with the fitted dose–response equally averaged over the two cities (i.e., using {− 1, + 1} as the city indicator variables). For this plot, the radiation dose scale is transformed by taking the square root to facilitate visualization of the low-dose range, although the fitted dose response and bootstrap confidence bands were computed on the untransformed dose scale.

## Discussion

This new and more thorough analysis of translocations in peripheral blood lymphocytes of atomic-bomb survivors exposed in utero supports the presence of an increase in translocation frequency in the dose region under 100 mGy despite the lack of an overall effect of radiation across the entire dose range. The overall dose response was not associated with whether individuals smoked at, or prior to, the time of blood collection. In addition to updating the analyses with the latest DS02R1 estimates of atomic-bomb radiation doses, we adjusted for urban–rural status at the time of exposure via adjustment for the interaction between city and proximal–distal location at the time of exposure. Nonparametric bootstrap confidence regions were newly added and these support the low-dose effect. It was difficult to precisely estimate the parameters of the low-dose effect due to the narrow dose range and large variability in observed translocation frequency. In addition, a gap in low-dose values in the study group made it difficult to pinpoint the location of the low-dose peak. Nevertheless, nonparametric smoothing reaffirmed the presence of a low-dose increase followed by a downturn, the likelihood surface for the low-dose parameters in the dose–response model was tractable, we were able to fit the model successfully using constrained maximum likelihood, and we ruled out undue influences of individual observations and parameter constraints. We were also able to confirm the result using an alternative fitting method, empirical Bayes estimation with Markov Chain Monte Carlo fitting. Although the estimated values of the low-dose parameters cannot provide precise estimates of low-dose risk because of uncertainty, there is little doubt of an increased risk of translocations at low doses.

The nonlinear model we used is expedient. It was designed to accommodate the conflicting observation of a lack of an overall radiation effect on lymphoid precursor cells despite a risk of childhood cancer (including leukemia) following in utero radiation exposure. It was also motivated in part by the nonparametric smoothing evidence, which suggested an increase restricted to low doses. We do not know whether it is the correct dose–response model; indeed, it does not estimate a precise peak location because of the global nature of the model and resulting restrictions imposed by it, but more detailed modeling of the low-dose hump is hampered by the narrow dose range and limited amount of data. However, it is noteworthy that the same low-dose increase was seen with nonparametric smoothing fits in all three separate trimesters of exposure. Because the trimesters are mutually exclusive, it is unlikely that the same pattern would occur in all three groups of participants if it were spurious. Therefore, although the true dose–response model could be different and the peak and spread are not precisely estimated, our model captures the qualitative pattern revealed by the data and is consistent with prior expectations of radiation effects on lymphoid precursor cells with in utero radiation exposure as discussed by Ohtaki et al. (2004).

In regression analyses one should try to avoid possible biases due to omitted variables. In observational studies, there is always the potential for confounding by unknown or unmeasured factors. With nonlinear models, there is also potential bias arising from omitted variables that are strongly associated with outcome (even though they are not associated with the exposure; see Cologne et al. 2019 for details and relevant references). Because geographic location is strongly associated with radiation dose in the atomic-bomb survivor studies, our model accounted for city and proximal–distal location at the time of atomic-bomb exposure, as well as the city–distance interaction (a potential surrogate for urban–rural status), as a means to address potential confounding related to location at the time of exposure (which could be associated with subsequent lifestyle and socio-economic factors that are risk factors for translocation induction). Given that in utero-exposed individuals were not born until after exposure, it is not clear to what extent such pre-bombing factors might act as confounders. We also specifically accounted for smoking, an established cause of translocations, without which there might possibly have been some omitted-variable bias. Although Sigurdson et al. (2008) demonstrated that smoking was associated with an increase in the upward curvature in translocation frequency with age, that effect is not relevant to our analysis because it occurred around age 60 in their study whereas all participants in our study had measurements at around the age of 40. The other potential cause of translocations, medical irradiation, could mimic a confounder by acting as a mediating variable; however, there was no noteworthy change in any of the basic atomic-bomb dose–response model parameters when potential effects of cumulative X-ray dose were simultaneously accounted for with analogous parameters (see the Online Resource, Sect. 5, Table S6).

Other factors that are potentially associated with translocation induction include race, gender, and exposure to environmental genotoxins. Gender was not strongly related to translocations in the analysis reported by Sigurdson et al. (2008); the female:male ratio of translocation proportion was 0.92 in their study (95% CI [0.83, 1.03]). The female–male difference in proportion in our analysis was 0.10% (± 0.05%, 95% CI [− 0.22, 0.12], with lower proportion in females). We also assessed whether there was an association between translocations and mother’s age near the time of conception, but found no evidence of such an association. The effect of father’s age at the time of conception could not be examined because many of the fathers were not identified in the data. Exposures to other clastogenic agents might be important, but we had no data on exposure to environmental agents other than smoking.

Important strengths of our investigation include the scoring of translocations in a laboratory renowned for its work in the field of chromosome aberrations in radiation-exposed populations (Kodama et al. 2001; Nakano et al. 2001), the availability of individual dose estimates that have been improved through careful assessment of location and shielding on top of already thorough dose reconstruction work (Cullings et al. 2017), and the conduct of sample and data collection in a controlled clinical setting. Strengths added in our detailed re-analysis include assessment of influence, examination of convergence via profile likelihoods, and verification of the basic dose–response model with an alternative (empirical Bayes) procedure.

Several limitations are worth noting. First, our analysis was based on a small sample size (330 participants). Statistical power is not an issue for the low-dose effect of radiation (since an effect was detected), but power could be responsible for lack of detecting an overall association between radiation and translocation frequency that manifests at higher doses. However, the estimated overall slope (about 1% per Gy with smoking adjustment) is far less than that for the mothers (a curvilinear increase, with an excess proportion of greater than 5% at 1 Gy based on Fig. 1 of Ohtaki et al, 2004), so it is unlikely that an effect as large as the one typically seen among atomic-bomb survivors was missed. Second, the in utero exposures here were from whole-body maternal exposure to radiation, not the abdominal or pelvic exposures that are common in most in utero-exposed cohorts. Third, we had no data on environmental exposures—apart from smoking—that could be associated with translocation induction (and hence a potential source of overdispersion). Omitting such variables from the model might result in omitted-variable bias in the estimated intercept, which in turn could affect the estimate of the low-dose slope. However, such bias could not conceivably alter the qualitative shape of the low-dose response unless the environmental exposures were strongly correlated with radiation doses in the low-dose range, which seems unlikely given the wide geographic distribution of persons who received low doses of atomic-bomb radiation. Fourth, it is possible that a greater number of extreme values of TF% were observed in the low-dose range, where there are more observations due to the skewed dose distribution. It is known that a larger sample size can result in larger dispersion because of the greater chance of observing extreme observations and the lower bound of zero on TF% could exaggerate this. However, there is no paucity of observations at extremely low doses or at zero dose, so any such effect caused by larger dispersion towards higher values of TF% should affect the intercept as well. Thus, it is unlikely that the low-dose increase in TF% is due to such a phenomenon. Fifth, there are no participants with doses between 2 and 20 mGy. We should therefore not rigidly interpret the location or spread of the low-dose peak, as it depends on the locations of the observed doses as well as on the form of the model. Nevertheless, a nonparametric smooth of the data above 20 mGy retains evidence of the low-dose hump despite the dose range lying above the location of the peak suggested by the data (see the Online Resource, Sect. 3.4, Fig. S8). Sixth, data on medical radiation exposures are subject to uncertainty. Radiation therapy was self-reported, although only one participant reported having received radiotherapy and it is unlikely that the participants of this study had received consequential exposures to radiation for therapeutic medical purposes given their ages at the time of the study. Diagnostic X-ray exposures were estimated from two sources: self-reported diagnostic X-rays received at outside institutions and radiographic and fluoroscopic procedures conducted at RERF (Yamamoto et al. 1988). Although the extent of random error in those assessments is unknown, the former were partly validated and the latter were strictly controlled. Seventh, fetal DS02R1 dose estimates are based on the uterine wall of a non-pregnant adult female; Paulbeck et al. (2019) note that this in general underestimates the fetal dose. Until new fetal dose estimates become available, however, we can only speculate that the location of the low-dose increase in translocation frequency might occur in a higher range of doses than that revealed in the present analysis.

Several topics could be candidates for future research, assuming that relevant data could be obtained. First, chromosome aberration frequency following radiation exposure might be affected by genetic factors, such as DNA repair gene polymorphisms (Djansugurova et al. 2020). We did not attempt to measure or adjust for any genetic factors as the blood samples were collected in the past for another purpose and informed consent was not obtained for genetic analysis. Such adjustment might help to reduce residual variability in the translocation frequencies and increase precision of the low-dose dose–response parameter estimates; this would be a worthwhile topic for future research. Second, we did not attempt to estimate a mediating effect of medical irradiation. Although medical irradiation for diagnosis and treatment typically involves small or localized doses, it is also associated with induction of peripheral blood lymphocyte chromosome aberrations (Shi et al. 2018; Shi and Tashiro 2018; Matsubara et al. 1974). Cumulative dose of medical irradiation for diagnostic or therapeutic purposes could be correlated with atomic-bomb radiation dose given that irradiated survivors might have had higher risks of diseases, such as cancer, that require irradiation for diagnosis or therapy (Sadakane et al. 2019). Medical irradiation could therefore be considered a potential mediator of the association between atomic-bomb radiation and subsequent lymphocyte TF. Mediation in causal models with data from observational studies has received a great deal of attention recently (VanderWeele 2015), so it should, in principle, be possible to assess mediation of an overall linear radiation effect across the entire dose range. However, the total overall radiation effect is close to null, so the proportion of overall increase that is mediated, no matter how large or small, would be difficult to estimate precisely. Nevertheless, because the low-dose part of the nonlinear model is the primary effect of interest, assessing mediation with that part of the model—and obtaining more precise estimates of doses of medical irradiation (as recommended by Sadakane et al. 2019)—would be a worthwhile goal for future research. Third, the association between chromosome aberrations and subsequent leukemia risk among the in utero-exposed persons would be an interesting topic of study. Although a follow-up analysis of only 330 individuals would probably not possess acceptable statistical power, it would be useful to ascertain subsequent lymphohematopoietic cancer incidence and mortality in relation to radiation dose received in utero. Such an analysis would best be served using updated dosimetry that includes specific fetal dose estimates, when that becomes available, rather than using the surrogate DS02R1 maternal uterus dose estimates.

## Supplementary Information

Below is the link to the electronic supplementary material.

Supplementary file1 (DOCX 3020 kb)

## Data Availability

The data analyzed in the current study are not publicly available due to policy of the Radiation Effects Research Foundation and legal privacy requirements of the Japanese Government. However, data can be obtained from the Radiation Effects Research Foundation upon reasonable request if the request is granted approval by the internal IRB.

## Code availability

Specialty code for the empirical Bayes analysis is provided in the Online Resource (Sect. 4). The entire reproducible research code and results are available, upon request to the first author, as HTML output created with knitr using Rmarkdown in RStudio.
