# Supplementary material for: Chromosome aberrations among atomic-bomb survivors exposed in utero: updated analysis accounting for revised radiation doses and smoking
Source: Radiat Environ Biophys. 2022 Feb 17;61(1):59–72. doi: 10.1007/s00411-021-00960-4 (PMC8897374; doi:10.1007/s00411-021-00960-4)
Supplement: Supplementary file 1 — Supplementary file1 (DOCX 3020 kb) [file 411_2021_960_MOESM1_ESM.docx]

ONLINE RESOURCE

Chromosome Aberrations Among Atomic-bomb Survivors Exposed In Utero: Updated Analysis Accounting for Revised Radiation Doses and Smoking

*Radiation and Environmental Biophysics*

John Cologne, Hiromi Sugiyama, Kanya Hamasaki, Yoshimi Tatsukawa, Benjamin French, Ritsu Sakata, Munechika Misumi

Corresponding author John Cologne, PhD, ELS

Department of Statistics

Radiation Effects Research Foundation

Email: [jcologne@rerf.jp](mailto:jcologne@rerf.jp)

**1. Additional data summaries**

The following tables show the distributions of mothers’ uterus doses (Table S1) and in utero survivors’ translocation proportions (TF/N; Table S2) according to category of in utero exposed participant’s smoking status.

**Table S1. Distribution of mothers’ uterus doses (mGy) by smoking status of in utero exposed survivor**

| **Quantile of dose** | **Never smoked** | **Current smoker** | **Past smoker** |
| --- | --- | --- | --- |
| 10% | 0.0 | 0.0 | 0.0 |
| 25% | 0.0 | 0.0 | 0.0 |
| 50% | 0.5 | 0.8 | 0.7 |
| 75% | 124.8 | 98.6 | 152.2 |
| 90% | 322.4 | 226.0 | 325.7 |
| 95% | 582.4 | 445.5 | 515.8 |
| 97.5% | 982.5 | 552.7 | 672.8 |

**Table S2. Distribution of in utero survivors’ translocation proportion by smoking status**

| **Quantile of translocation proportion** | **Never smoked** | **Current smoker** | **Past smoker** |
| --- | --- | --- | --- |
| 10% | 0 | 0 | 0 |
| 25% | 0 | 0.010 | 0 |
| 50% | 0.010 | 0.010 | 0.010 |
| 75% | 0.020 | 0.020 | 0.020 |
| 90% | 0.030 | 0.030 | 0.030 |
| 95% | 0.040 | 0.040 | 0.040 |
| 97.5% | 0.040 | 0.050 | 0.042 |

A lack of clear differences by smoking category in either dose or translocation proportion suggests lack of confounding by smoking.

The following histogram (Figure S1) depicts the distribution of mothers’ ages at the time of the bombing (which was used as a surrogate for mother’s age at the time of conception).


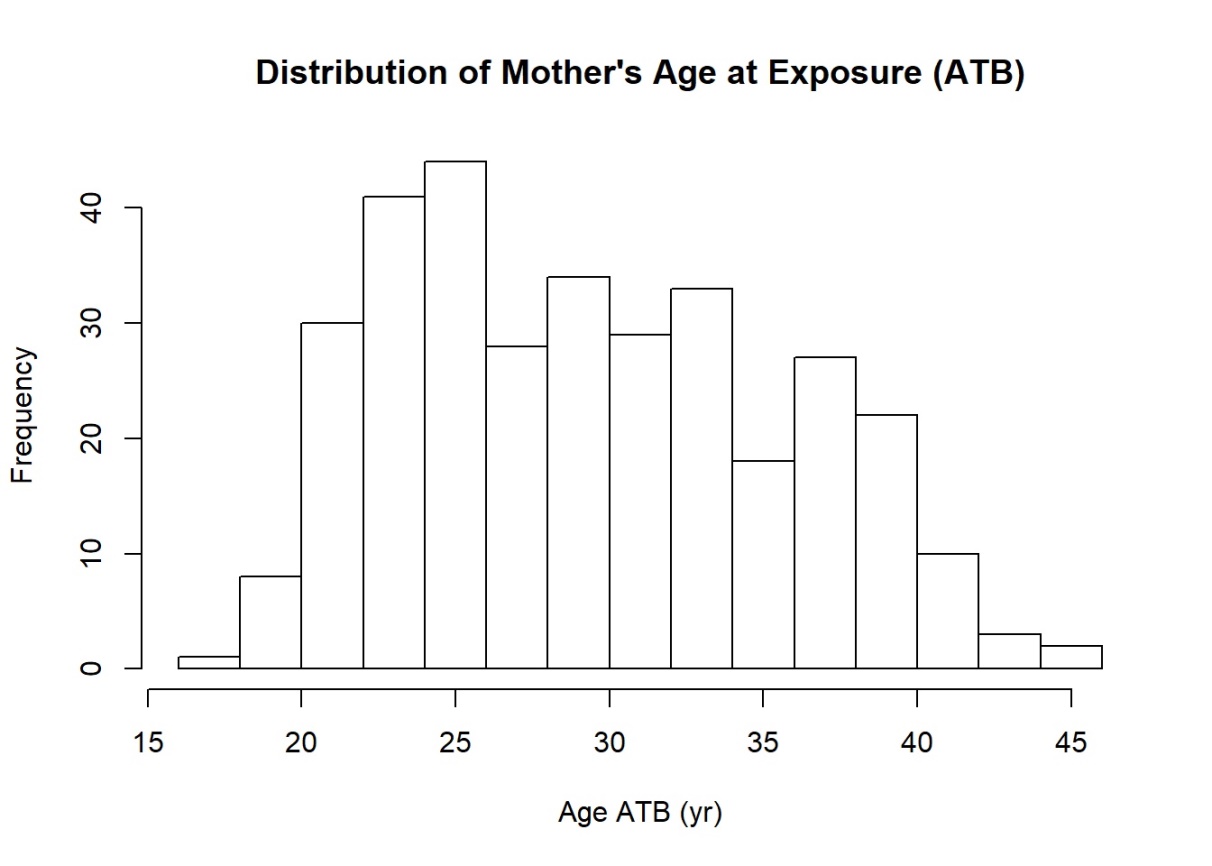


**Figure S1. Distribution of mothers’ ages at the time of A-bomb radiation exposure.**

**2. Nonparametric smoothing**

The following plots (Figure S2) show the results of nonparametric smoothing applied to scatterplots of translocation proportion versus DS02R1. Plotting and smoothing (solid lines) were performed on the square-root scale for observations with doses below 200 mGy to allow better visualization of the dose response at low doses and to reduce the impact of skewness on the smoothing algorithm. Smooths made on the untransformed scale are shown as dashed lines.

Smooths were obtained with super smoother (R function **supsmu**), with smoothing span selected by cross-validation (the default approach) and the bass parameter set to 10 (bass 10 imposes the maximum possible amount of smoothness). Plots are shown for all trimesters combined (upper left) and for each of the three separate trimesters at the time of exposure (reading across from left to right, then down). Estimated trimester of exposure was updated from the original analysis by Ohtaki et al (2004), as described in the Methods section of our manuscript.


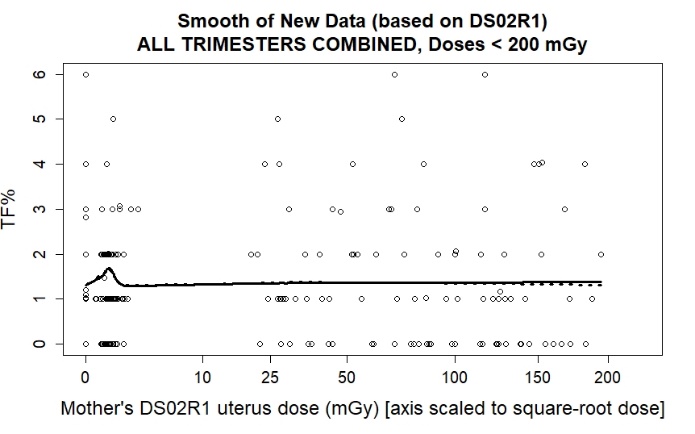

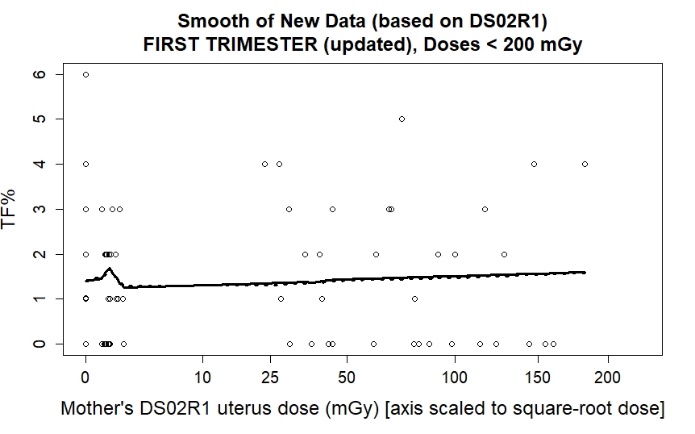


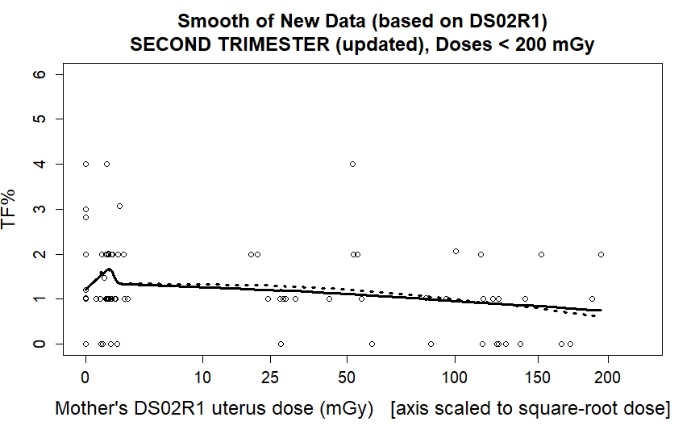

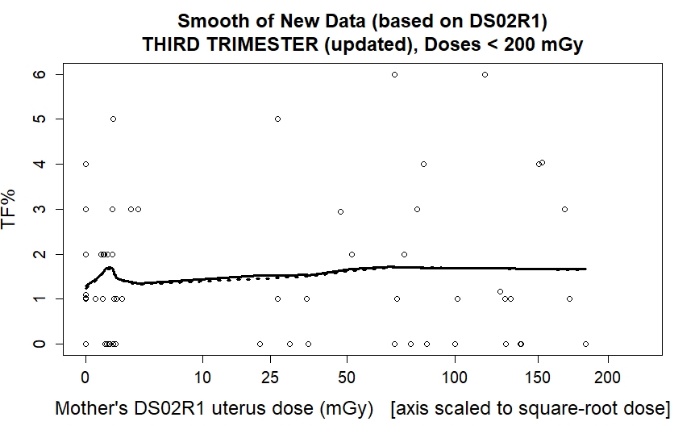


**Figure S2. Nonparametric smooths of the chromosome aberration (translocation) frequency data (number of aberrations per 100 cells scored; %) as a function of mother’s DS02R1 uterus dose.**

There is an apparent increase at low doses followed by a decline, as was seen with DS86 in the original analysis by Ohtaki et al (2004). Furthermore, the low-dose increase is seen for each of the three mutually exclusive subsets based on trimester. As mentioned in the main paper, there is a gap with no observations at doses between 2.1 and 20.0 mGy. Smoothing on the original dose scale, rather than the square-root scale, had little effect, as shown by the dashed lines. It is not possible to infer from these smooths precisely where the peak and downturn occur. For this reason, the location of the peak in the curves fitted with the nonlinear model in the main paper should be interpreted with caution as it will be determined not only by the data but also by the shape of the nonlinear function.

**3. Sensitivity analyses**

*3.1. Sensitivity to non-negativity constraints*

Although it seems natural to constrain the intercept to be non-negative to avoid a negative estimate of the binomial proportion, it could be argued that the constraints might not hold in theory for the three dose-related parameters of the basic dose-response model (initial slope, downturn, and overall slope). When the overall intercept was not constrained, convergence was extremely sensitive to starting values and it was not possible to obtain bootstrap confidence intervals because the unconstrained optimization did not converge with some bootstrap samples due to negative fitted values of the binomial proportion. We therefore fit the seven-parameter model (basic four-parameter model with city-distance terms added) without constraints on any parameter except the overall intercept. The following table (Table S3) shows the results without constraints alongside the results with constraints (from Table 3 in the main paper).

Table S3. Parameter estimates (95% confidence intervals) for the dose-response model ^a^ with city-distance terms, without or with non-negativity constraints on the dose-related parameters

| Parameter | Without constraints  Loglik ^b^ = −500.92 | With constraints ^c^  loglik = −500.91 |
| --- | --- | --- |
| *θ* _1_ — intercept (%) | 1.51  (0.97, 2.25) | 1.50  (0.94, 1.74) |
| *θ* _2_ — initial slope (%/mGy) | 0.013  (−0.11 0.47) | 0.016  (0.0, 0.11) |
| *θ* _3_ — downturn (mGy^−1^) | 0.043  (0.014, 0.13) | 0.048  (0.027, 0.052) |
| *θ* _4_ — overall slope (%/Gy) | 0.071  (−1.24, 1.30) | 0.071  (0.0, 1.51) |
| *θ* _5_ — city (Δ%) ^c^  (coded as ± 1) | 0.095  (−0.24, 0.43) | 0.094  (−0.21, 0.40) |
| *θ* _6_ — distal (Δ%) | −0.26  (−1.1, 0.25) | −0.26  (−0.48, 0.29) |
| *θ* _7_ — Nagasaki-distal interaction (Δ%) | 0.16  (−0.61, 0.97) | 0.17  (−0.67, 0.91) |
| ^a^ The model is $p_{\theta}=\theta_{1}+\left[\theta_{2}d\mathrm{e}^{-\theta_{3}d}+\theta_{4}d\right]+\theta_{5}c+\theta_{6}g+\theta_{7}(n\times g)$ where *d* is mother’s uterus radiation dose, *c* is city (coded ±1), and *g* is distal ground distance (≥ 3 km)  ^b^ log-likelihood  ^c^ Non-negativity constraints were applied to the initial slope, downturn, and overall slope parameters (in addition to the intercept); city-distal parameters were not constrained (results with constraints are those in Table 3 of the main paper) | | |

The fitted parameters are nearly identical without or with constraints. Without constraints, the lower confidence bounds on the initial slope and overall slope are negative, and the uncertainty in the intercept is larger than when the dose-related parameters are constrained to be non-negative. The low-dose increase over a restricted dose range does not appear to be an artifact of the non-negativity constraints because, without constraints, the downturn parameter still has a lower confidence bound that exceeds zero.

*3.2. Influence assessment*

Influence of individual observations was examined with the jackknife-after-bootstrap method (Efron, 1992; Davison and Hinkley, 1997) for the seven-parameter model of Table 3 of the main paper (left column; the basic four-parameter dose response with the three city-distance terms added). The procedure takes advantage of the fact that the bootstrap routine records which observations were resampled in each iteration of the bootstrap, so it is straightforward to compute percentiles of the bootstrap distribution for all iterations where a particular sample did not appear (similar to leave-one-out influence calculations that are often applied in ordinary regression diagnostics). Below (Figure S3) are plots produced by applying the **jack.after.boot** function in the R **boot** package to each of the seven parameters. The **jack.after.boot** function plots several percentiles of the bootstrap distribution from all iterations where the random sample did not contain a particular observation, repeatedly for each of the 330 observations in the data. The left-out observations are ordered according to the estimated influence function, and the ID number of the observation is printed at the bottom of the plot. (Random ID numbers were assigned to all observations in our study.)

None of the plots suggests serious influence (a more pathologic example can be seen in the example provided by Efron, 1992). The initial slope (parameter 2) and the overall slope (parameter 4) show slight (though not troublesome) influence of a few observations as judged by expansion or contraction of the percentiles at either the left or the right extreme of the horizontal axis, so we assessed influence on these two parameters in greater detail.


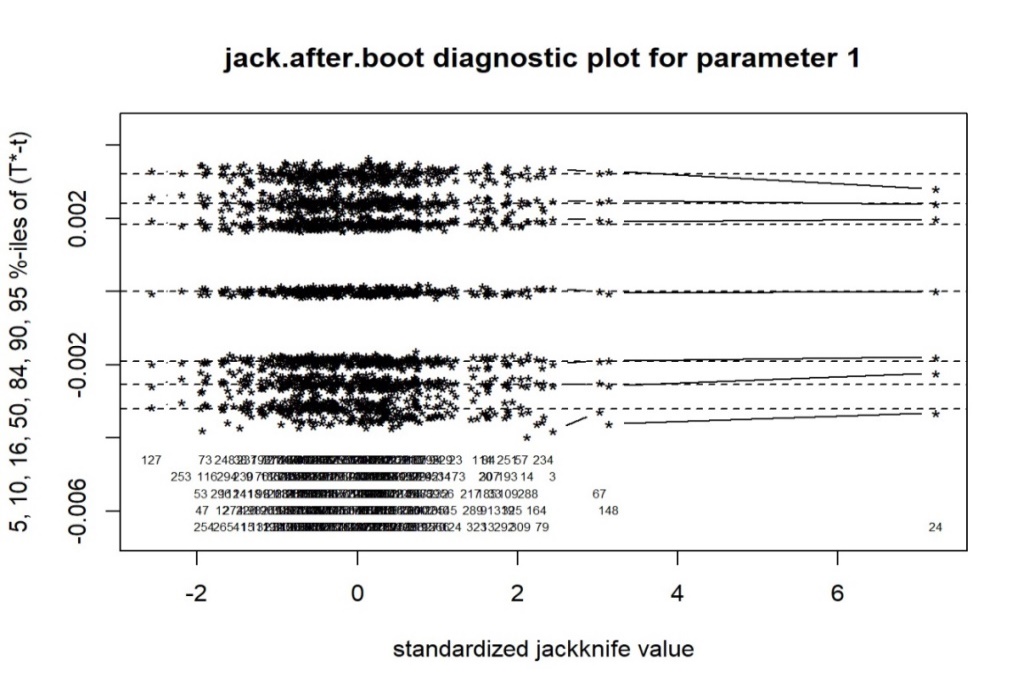

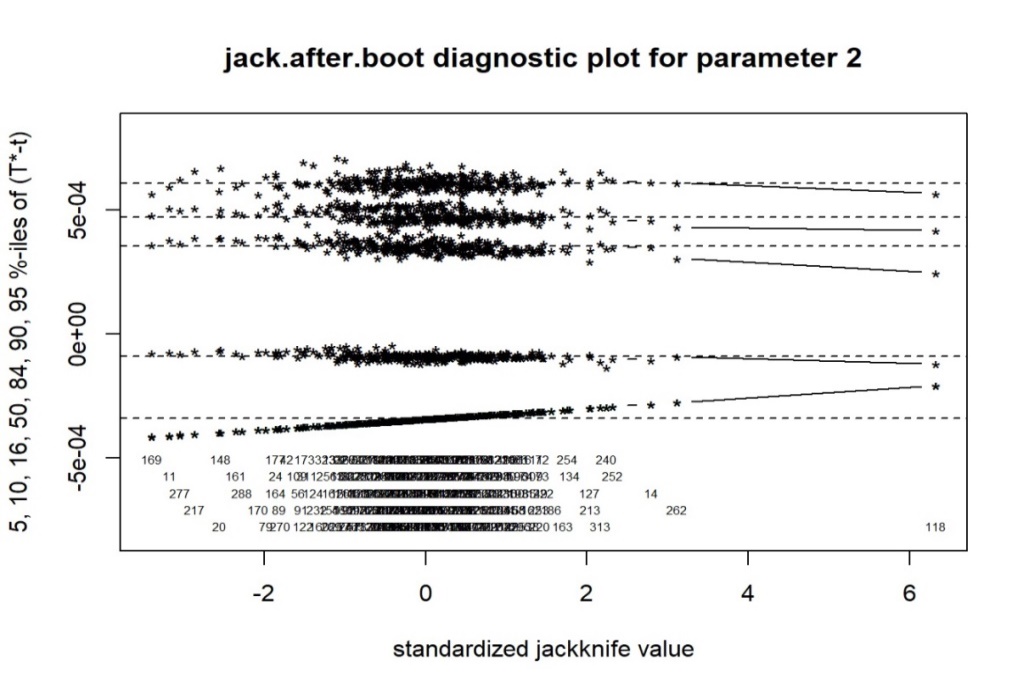

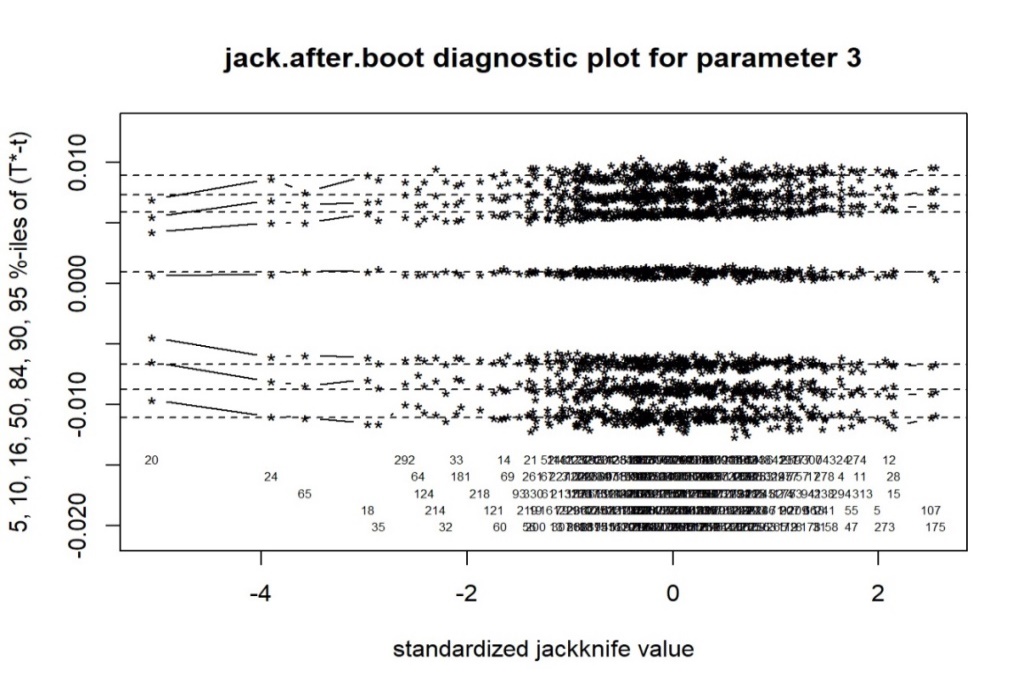

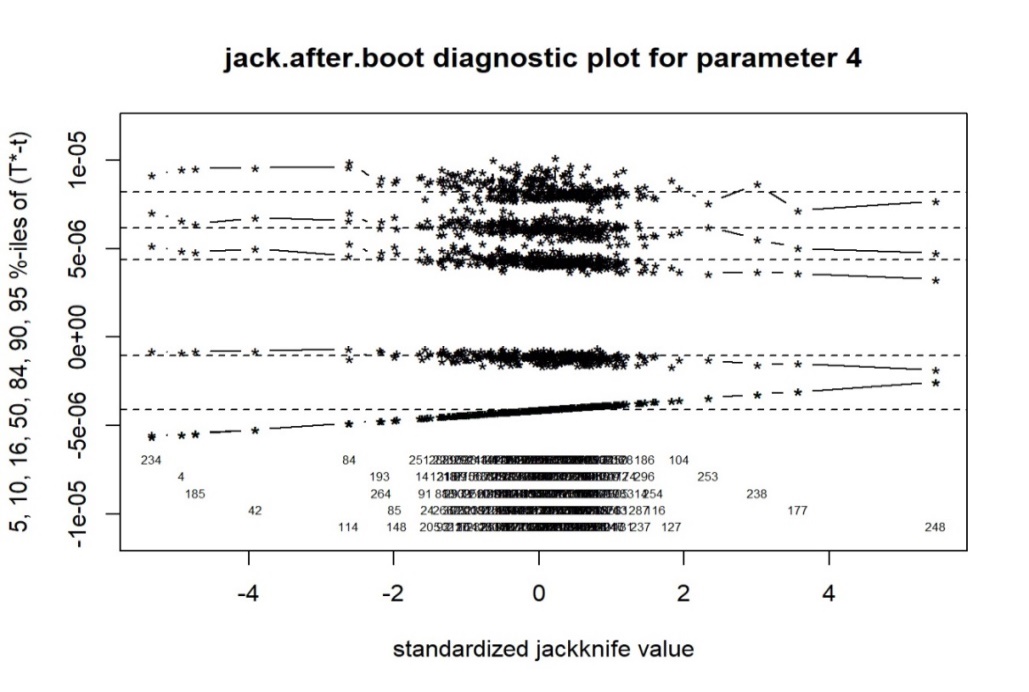


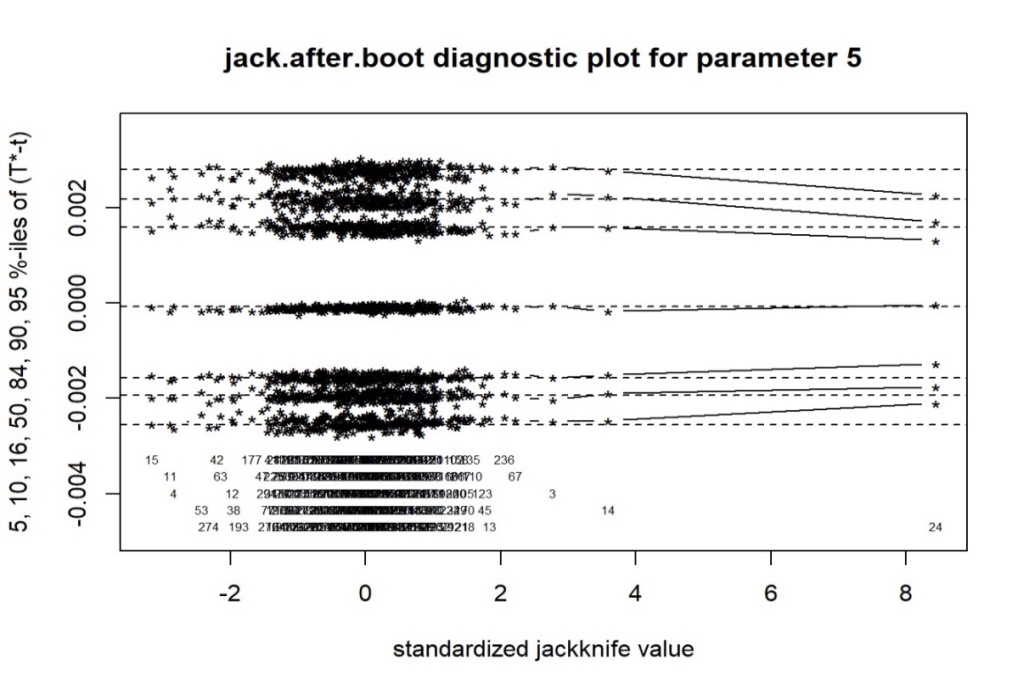

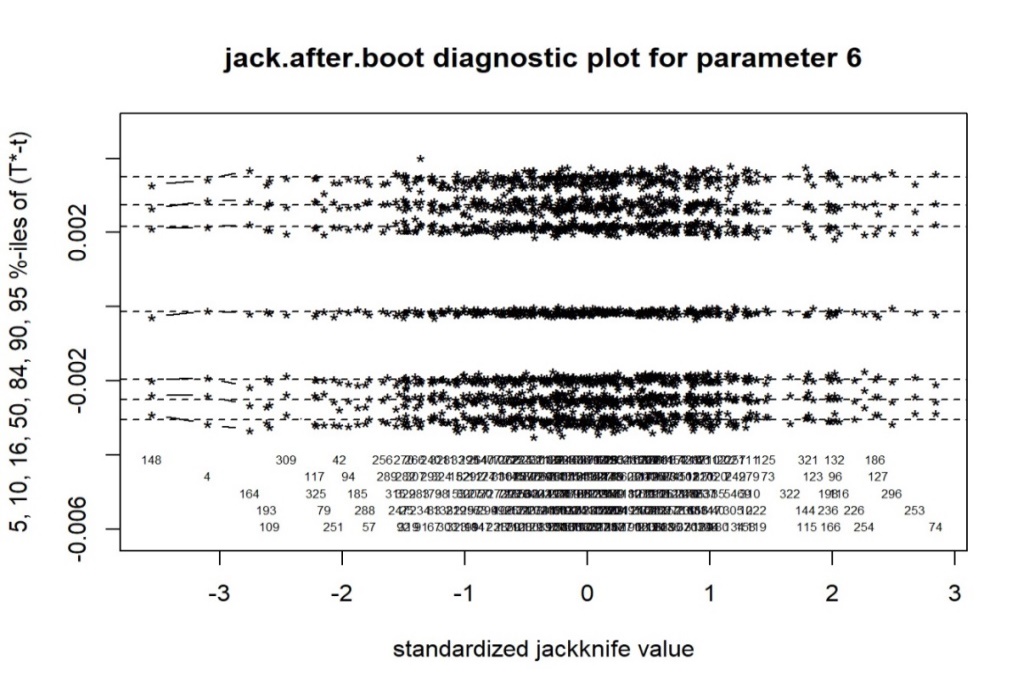

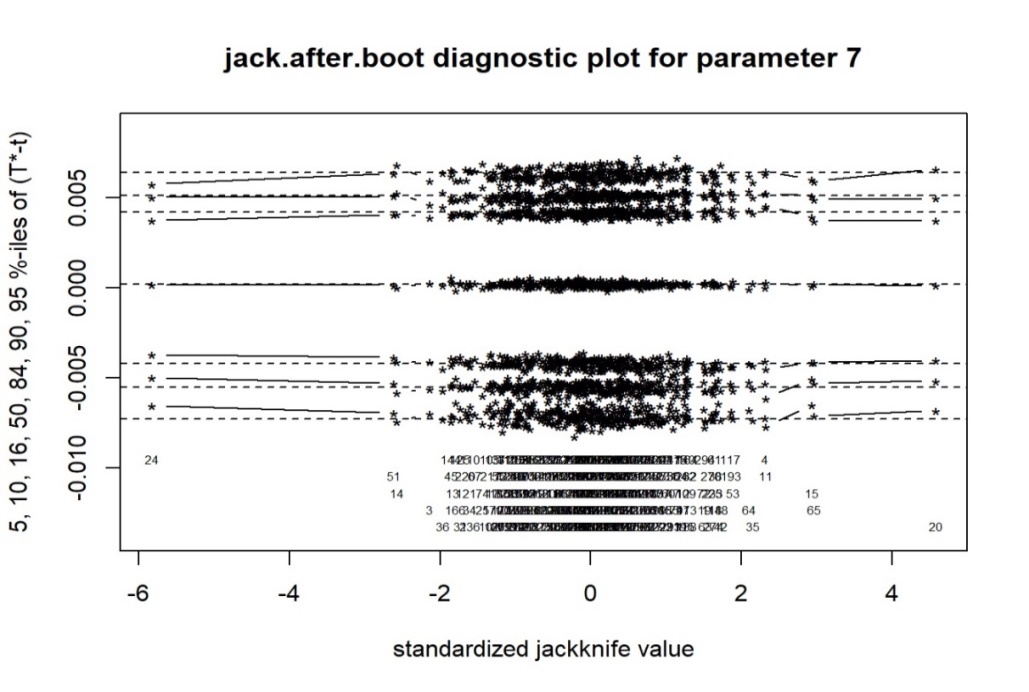


**Figure S3. Jackknife-after-bootstrap plots showing influence of individual observations on parameters 1−7 of the seven-parameter model. Horizontal lines represent the mean levels at the 5th, 10th, 16th, 50th, 84th, 90th, and 95th percentiles of the bootstrap distribution with each individual observation omitted.**

Although none of the parameters exhibits unreasonable influence due to any of the observations (as indicated by generally horizontal trends in the above plots), we nevertheless followed up on the potentially most influential points. Two points appeared to have some influence on the initial slope (parameter 2). These are shown on the following plot (Figure S4) in green (point 118) and in red (point 11). The plot focuses on the low-dose range (up to 40 mGy).


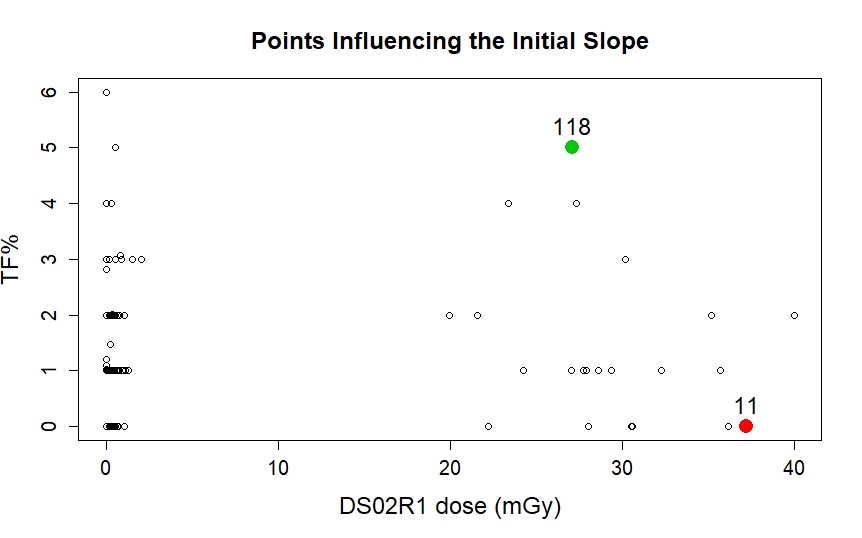


**Figure S4. Plot of the data in the region 40 mGy and below identifying observations, revealed by the jack-after-boot procedure, that had especially large influence on the initial dose-response slope.**

Following (Table S4) are results obtained with these two points excluded one-at-a-time from the constrained maximum likelihood fits. To facilitate comparison, we include the results, from Table 3 of the main paper, that were obtained with all observations.

**Table S4. Parameter estimates (95% bootstrap confidence intervals) for the dose-response model of Table S3 (with city and distance added), with omission of observations influencing the initial slope**

| Parameter | Observation excluded | | |
| --- | --- | --- | --- |
|  | #11  loglik ^a^ = −499.14 | All data  loglik = −500.91 | #118  loglik = −498.75 |
| Intercept (%) | 1.53  (1.01, 1.79) | 1.50  (0.94, 1.74) | 1.40  (0.99, 1.72) |
| Initial slope (%/mGy) | 0.023  (0.0, 0.13) | 0.016  (0.0, 0.11) | 0.035  (0.0, 0.099) |
| Downturn (mGy^−1^) | 0.047  (0.026, 0.052) | 0.048  (0.027, 0.052) | 0.039  (0.028, 0.052) |
| Overall slope (%/Gy) | 0.069  (0.0, 1.41) | 0.071  (0.0, 1.51) | 0.53  (0.0, 1.43) |
| City (Δ% ^b^)  (coded as ± 1) | 0.13  (−0.20, 0.44) | 0.094  (−0.21, 0.40) | 0.27  (−0.19, 0.41) |
| Distal (Δ%) | −0.25  (−0.51, 0.30) | −0.26  (−0.48, 0.29) | 0.075  (−0.48, 0.28) |
| Nagasaki-distal interaction (Δ%) | 0.096  (−0.66, 0.91) | 0.17  (−0.67, 0.91) | 0.43  (−0.63, 0.93) |
| ^a^ log-likelihood  ^b^ Δ% is difference from overall mean in translocation percentage; Hiroshima coded −1, Nagasaki coded +1 | | | |

It is difficult to find any pattern in the influence of these two points. Both of them apparently tended to cause a lower estimate of the initial slope (i.e., their elimination resulted in higher estimates of the initial slope). This might be because of the proximity of the low-dose range to the intercept; any point that influences either will probably influence both, so it might be difficult to predict what the effect of a point will be. Point #118 apparently had a lowering influence on the overall slope, presumably by increasing the mean TF% in the region close to the intercept (indeed the estimated intercept was lower when #118 was removed).

Several observations appeared to have some influence on the overall slope. Again, these are shown in green (point 248) or red (point 185). The plot (Figure S5) covers the entire dose range.


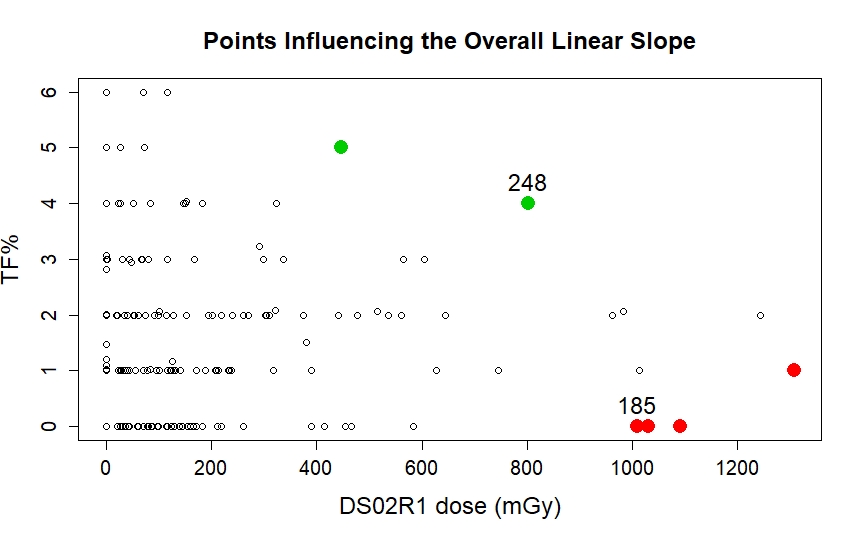


**Figure S5. Plot of the data identifying observations, revealed by the jack-after-boot procedure, that had especially large influence on the overall dose-response slope.**

Following (Table S5) are results obtained with two of these points excluded one-at-a-time from the constrained maximum likelihood fit.

**Table S5. Parameter estimates (95% bootstrap confidence intervals) for the dose-response model of Table S3 (with city and distance added), with omission of observations influencing the overall slope**

| Parameter | Observation excluded | | |
| --- | --- | --- | --- |
|  | #185  loglik ^a^ = −499.35 | All data  loglik = −500.91 | #248  loglik = −497.71 |
| Intercept (%) | 1.46  (0.90, 1.73) | 1.50  (0.94, 1.74) | 1.50  (0.99, 1.73) |
| Initial slope (%/mGy) | 0.021  (0.0, 0.13) | 0.016  (0.0, 0.11) | 0.019  (0.0, 0.11) |
| Downturn (mGy^−1^) | 0.046  (0.028, 0.052) | 0.048  (0.027, 0.052) | 0.047  (0.029, 0.052) |
| Overall slope (%/Gy) | 0.25  (0.0, 1.69) | 0.071  (0.0, 1.51) | 0.000005  (0.0, 1.43) |
| City (Δ% ^b^)  (coded as ± 1) | 0.083  (−0.22, 0.40) | 0.094  (−0.21, 0.40) | 0.11  (−0.18, 0.39) |
| Distal (Δ%) | −0.23  (−0.51, 0.32) | −0.26  (−0.48, 0.29) | −0.25  (−0.50, 0.27) |
| Nagasaki-distal interaction (Δ%) | 0.19  (−0.66, 0.91) | 0.17  (−0.67, 0.91) | 0.14  (−0.60, 0.89) |
| ^a^ log-likelihood  ^b^ Δ% is difference from overall mean in translocation percentage; Hiroshima coded −1, Nagasaki coded +1 | | | |

Point #185 has leverage on the overall slope and, with a TF% value of zero, tends to cause a lower estimate of the overall slope; deleting #185 results in a somewhat higher overall slope estimate. Point #248, on the other hand, had a large TF% value, so with its leverage it causes a higher estimate of the overall slope (i.e. deleting #248 results in a much lower overall slope estimate).

*3.3. Convergence assessment via profile likelihood*

Low-dose parameters in basic dose-response model. The following plot (Figure S6) is of contours of the profile likelihood for the initial slope (*θ*_2_) and exponential downturn (*θ*_3_) in the basic four-parameter dose-response model with DS02R1. A 50×50 grid of fixed and equally spaced points was used. Convergence to the maximum log-likelihood solution was achieved at all 2,500 points. The maximum value of the log-likelihood is −503.18, the same as the maximum log-likelihood value from the constrained maximum likelihood estimation (MLE; lower part of Table 2 of the main paper). However, the values of the two parameters at the maximum profile likelihood (marked as “X”) are slightly different from those obtained with constrained MLE (the green dot). We attribute this to subtle differences in how the fitting algorithm seeks to converge towards a solution of the other two parameters (the **maxLik** function may choose an appropriate numerical algorithm and perform step-halving as needed). In addition, it has been noted that profile likelihood can produce misleading inference in “certain situations” (Li GY: *Statistical Analysis with Measurement Error or Misclassification*; Springer, 2017, page 22), so we do not consider the inconsistency to be cause for concern.


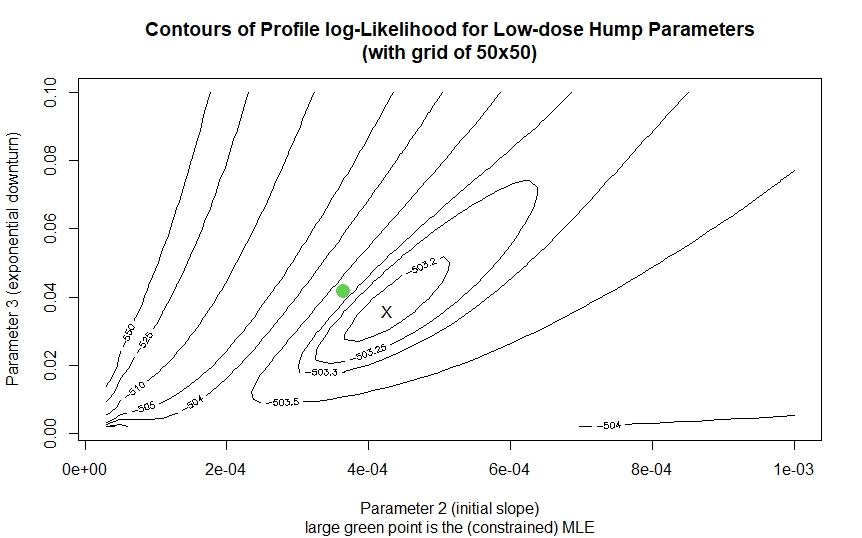


**Figure S6. Profile log-likelihood contours for the two low-dose parameters in the basic four-parameter dose-response model.**

That the exponential downturn parameter is not defined when the initial slope is zero is evident from the asymmetric behavior of the profile likelihood near the origin. However, the region near the maximum log-likelihood comprises nearly symmetric ovals, which means that a solution exists and should be found as long as reasonable starting values are used. The primary message is that the profile likelihood supports the constrained maximum likelihood fit of the model.

Smoking-behavior parameters. The following plot (Figure S7) shows profile likelihood contours for the two smoking-behavior parameters added to the intercept of the city-distance adjusted dose-response model. A 3×3 grid is used because finer grids produced a great deal of noise (more is said about this below). Convergence of the constrained MLE for the other parameters was achieved at all nine points on the grid.


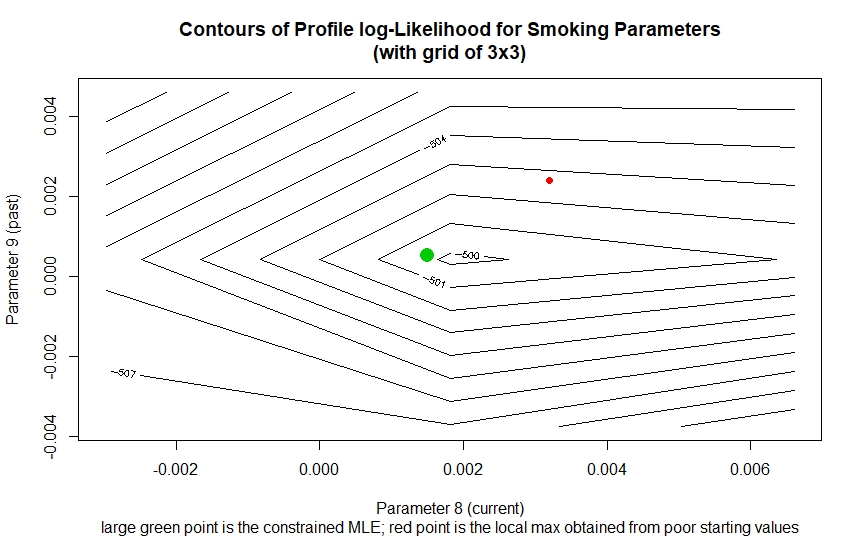


**Figure S7. Profile log-likelihood contours for the two smoking-behavior parameters in the expanded dose-response model (left column of Table 4 in the main manuscript, without smoking-radiation interaction).**

The plot shows well-behaved concentric contours with a maximum log-likelihood of −499.78, slightly different from the value −502.98 for the maximization of all parameters jointly (the MLE parameter estimates are shown by the green point). The reason for using a coarse (3×3) grid is that, with finer grids, there is erratic behavior with distortions in the contours resulting in many local maxima. These are probably due to the discrete nature of the smoking variables in the contours. Indeed, when very large starting values, far from the global maximum, were used, the constrained MLE converged to the red point far from the peak of the profile likelihood.

*3.4. Nonparametric assessment of dose-response above 20 mGy*

As noted in the main manuscript, there are no participants with doses between 2 and 20 mGy. A reviewer suggested examining the translocation dose response above 20 mGy. Because 20 mGy is a higher dose than the apparent location of the peak suggested by the data in the nonparametric smooths, the nonlinear dose-response model cannot be fit to the dose range above 20 mGy because the baseline aberration frequency (the frequency at 20 mGy) would lie on the downturn part of the low-dose hump. However, the nonparametric smoother (R function **supsmu**) applied to the dose range above 20 mGy — and imposing the maximum possible amount of smoothness — reveals that there is still evidence of the low-dose increase in translocation frequency, as shown in the following plot (Figure S8).


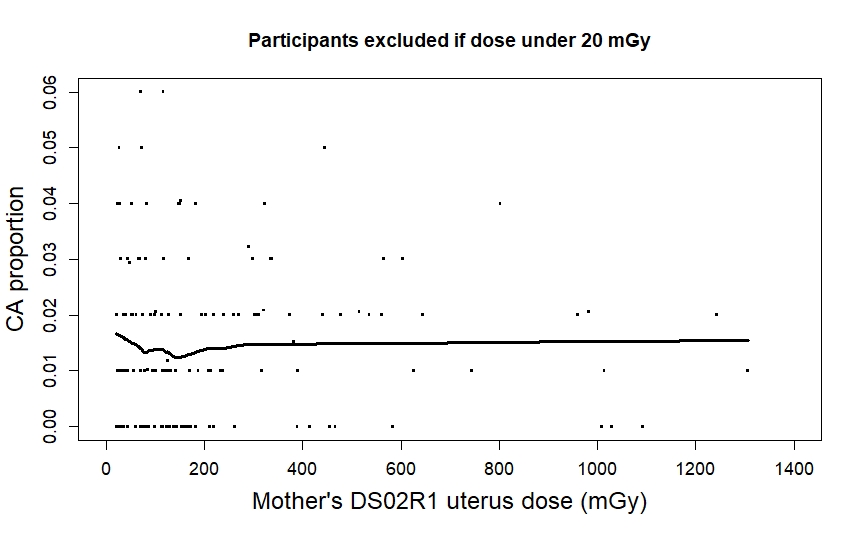


**Figure S8. Nonparametric smooth of the chromosome aberration (translocation) frequency data (number of aberrations per 100 cells scored; %) as a function of mother’s DS02R1 uterus dose, with data restricted to the dose range above 20 mGy.**

We do not ascribe any meaning to the fitted smooth above about 200 mGy because it is heavily influenced by the small number of points at the higher end of the dose range and is therefore subject to large variability.

**4. Empirical Bayes fit of basic 4-parameter dose-response model**

Here we provide the results from fitting the basic 4-parameter dose response with an empirical Bayes approach using Markov Chain Monte Carlo simulation to estimate the posterior distributions of the four parameters. Diffuse prior distributions were used for each of the parameters: uniform for the intercept (*θ*_1_) and the two slope parameters (initial slope *θ*_2_ and overall slope *θ*_4_), and normal for the exponential downturn *θ*_3_; these prior distributions were set to be widely dispersed relative to the standard errors of the parameter estimates obtained by constrained maximum likelihood (see the Online Resource for details). Posterior distributions were sampled from 1.5×10^6^ iterations after a burn-in of 5×10^6^ iterations, which was determined through trial and error by examination of trace plots of chain iterations for all four parameters as well as the deviance. After the burn-in, simulated values were estimated from 500,000 iterations on each of three independent chains (total of 1,500,000 simulated values), with each chain having a different starting value.

The BUGS code (fit via the BRugs package in R) is

model_string <- "

model

{

for (i in 1:Nrecs) {

p[i] <- theta1 + theta2*dose[i]*exp(-theta3*dose[i]) + theta4*dose[i]

CAfreq[i] ~ dbin(p[i], N[i])

}

theta1 ~ dunif(0,.03)

theta2 ~ dunif(0,.006)

theta3 ~ dnorm(.04,10000)

theta4 ~ dunif(0,.00005)

}

"

writeLines(model_string, con="basicMCMCmodel.txt")

model_inits1 <- "

list(theta1=.01, theta2=.0003, theta3=.035, theta4=.000002)

"

writeLines(model_inits1, con="basicMCMCinits1.txt")

model_inits2 <- "

list(theta1=.015, theta2=.0004, theta3=.04, theta4=.000004)

"

writeLines(model_inits2, con="basicMCMCinits2.txt")

model_inits3 <- "

list(theta1=.02, theta2=.0005, theta3=.045, theta4=.000006)

"

writeLines(model_inits3, con="basicMCMCinits3.txt")

model_data <- list(Nrecs=length(CAfreq), CAfreq=CAfreq, N=N, dose=dose02R1)

bugsData(model_data, fileName="basicMCMCdata.txt", format="fg")

modelCheck("basicMCMCmodel.txt")

modelData("basicMCMCdata.txt")

modelCompile(numChains=3)

modelInits("basicMCMCinits1.txt", chainNum=1)

modelInits("basicMCMCinits2.txt", chainNum=2)

modelInits("basicMCMCinits3.txt", chainNum=3)

modelUpdate(5000000)

samplesSet(c("theta1", "theta2", "theta3", "theta4", "deviance"))

modelUpdate(1500000)

samplesStats(c("theta1", "theta2", "theta3", "theta4", "deviance"))

samplesHistory("theta1", mfrow=c(1,1), ask=F)

samplesHistory("theta2", mfrow=c(1,1), ask=F)

samplesHistory("theta3", mfrow=c(1,1), ask=F)

samplesHistory("theta4", mfrow=c(1,1), ask=F)

samplesHistory("deviance", mfrow=c(1,1), ask=F)

samplesCorrel("theta2", "theta3")

Uniform priors were assigned to the intercept (parameter theta 1), initial slope (parameter theta 2), and overall slope (parameter theta 4), with ranges from 0 to (0.03, 0.006, 0.00005) respectively (using zero as the lower bound on the uniform prior mimics the constraints applied in the constrained maximum likelihood fits). The upper limits on these three uniform priors were set so that they were at least as high as the value of the corresponding estimate plus ten times its standard error from the constrained maximum likelihood fit. Note that the parameters were estimated on the scale of proportion for the outcome and mGy for dose, as with the constrained MLE fits in the main paper, so the scales of parameters 1 and 2 are 0.01, and that of parameter 4 0.00001, relative to values reported in tables in the main text. A normal prior with mean 0.04 and precision 10,000 was assigned to the exponential downturn parameter (parameter theta3). This precision gives 97.5% of normal distribution of .0596, close to the bootstrap 95% confidence interval upper bound of 0.05735 (this can be confirmed by running qnorm(.975, .04, sqrt(1/10000)) in R). Although this upper limit is not so diffuse, it was necessary to achieve convergence with parameter theta3.


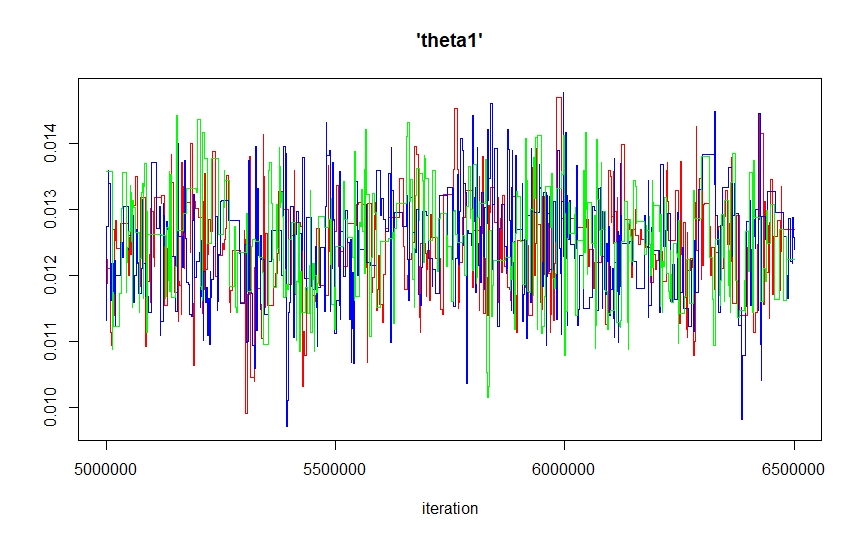


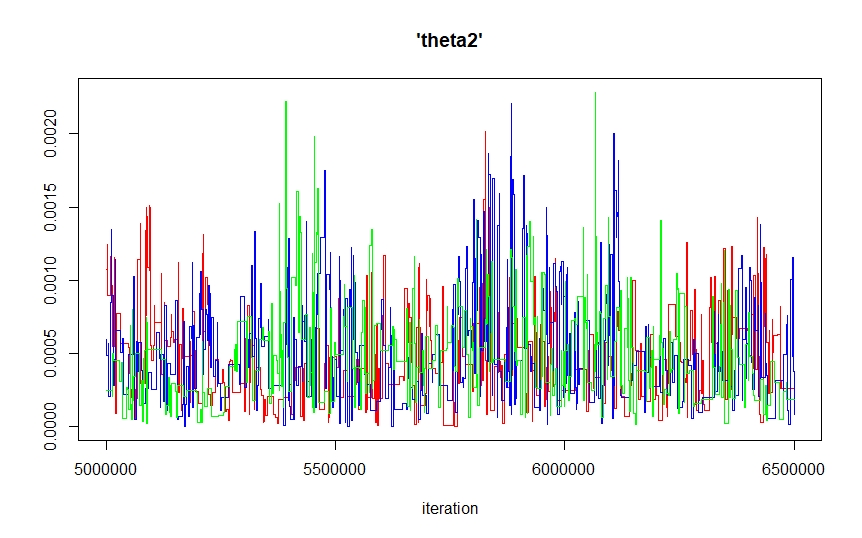


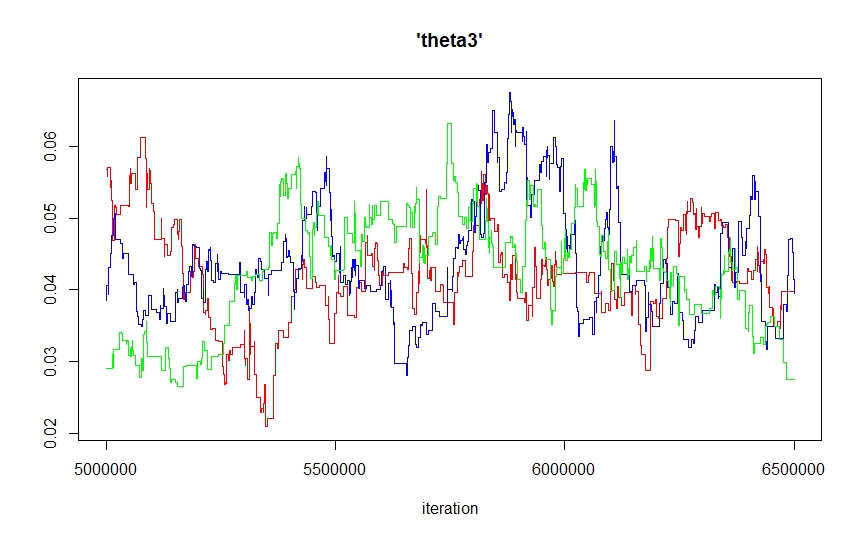


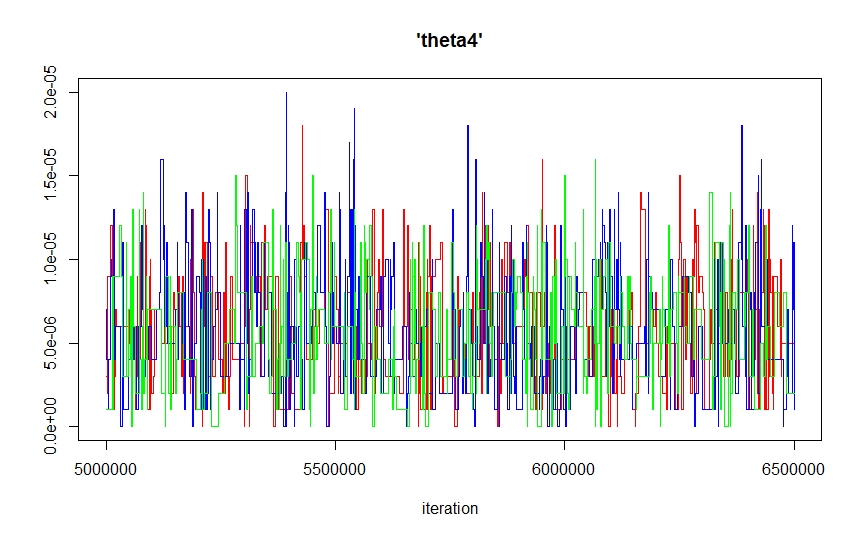


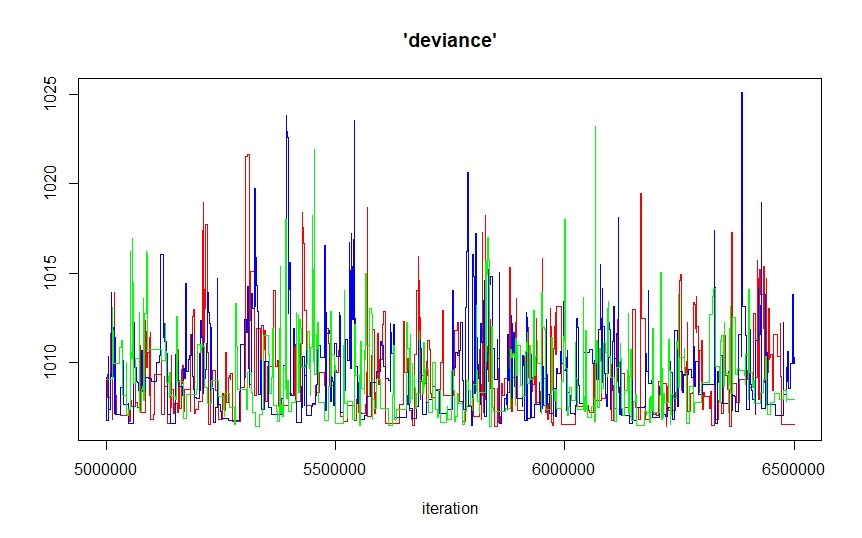


The burn-in appears to have been sufficient for all parameters except for the exponential downturn. All parameters except for the downturn appear to have converged, as judged by the random and stable iteration tracings and the ample mixing of the three simulation chains. The exponential downturn is difficult to estimate, as noted earlier in the discussion of the profile likelihood for the two low-dose parameters. This is apparently also the reason for the occasional spikes in the tracings for the deviance. The median values of the four parameters were: intercept, 1.26%; initial slope, 0.0412 %/mGy; downturn, 0.0422; overall slope, 0.528. These are close to the constrained MLE values with DS02R1 (lower part of Table 2 of the main paper). The median deviance was 1,008, not far from twice the value of the log-likelihood at the constrained MLE (1,006), although these two values are not directly comparable given that the empirical Bayes approach maximizes the posterior likelihood based on the prior densities for the parameters, not the binomial likelihood alone.

**5. Addition of medical X-ray doses to the basic 4-parameter dose-response model**

Here we provide results from fitting the basic 4-parameter dose response model for atomic-bomb exposure with adjustment for estimated medical diagnostic X-ray doses. Because persons exposed to atomic-bomb radiation might be at higher risk for cancer and other chronic diseases, it is conceivable that the extent of subsequent medical procedures involving radiation (diagnostic and therapeutic) after birth (especially during adulthood) could be correlated with atomic-bomb dose. As mentioned in the main manuscript, only one participant in the present study was known to have received radiation therapy, but estimates of cumulative diagnostic X-ray exposure were available for all participants. Estimates of external X-ray doses ascertained through interviews at the biennial clinical examinations were combined with estimated X-ray doses delivered during those examinations, as reported by Yamamoto et al. (1988). We use the simple average of bone marrow and gonad dose estimates in mGy. The minimum mean estimated cumulative X-ray dose was 1.41 mGy and the maximum was 744.1 mGy. Although the distribution of these average estimated doses was skewed, we did not log-transform them so that we could apply them in the analysis with the same form of dose-response model as was used for in utero atomic-bomb exposure to assess the sensitivity of all parameters of the atomic-bomb dose-response model.

We are not aware of a method for dealing with mediation in a complex nonlinear model, such as the dose-response model used in the present work. We also do not know how potential mediation by medical X-rays might affect the parameters of the atomic-bomb dose response. We therefore adjusted for estimated X-ray dose as if it were a confounder (a factor that is potentially causally related with the outcome — TF% — and that is correlated with the exposure of interest — atomic-bomb radiation dose), although in the case of mediation the intermediate factor (X-ray dose) occurs after, and is potentially caused by, the exposure, not vice-versa as in the case of confounding. To assess the influence of estimated X-ray dose on the atomic-bomb dose-response parameter estimates, we adjusted for X-ray dose using either the same three types of dose-related parameters as in the atomic-bomb dose response model or a simple linear model for the effect of X-ray dose. The former might be considered the most sensitive adjustment, allowing each estimated parameter of the atomic-bomb dose response to be influenced by an analogous term involving X-ray dose. The latter might be considered the more appropriate and standard type of adjustment, given that X-ray doses received during adulthood would not be expected to cause the same type of dose-response as was observed for fetal exposure atomic-bomb radiation. We also attempted to fit a model with linear-quadratic adjustment for X-ray dose, but were unable to get the estimates of such a model to converge.

The table below (Table S6) shows parameter estimates obtained from the fit of the basic dose-response model with separate parameters for atomic-bomb dose and estimated medical diagnostic X-ray dose, where the model for X-ray dose mimics that for atomic-bomb radiation dose. In other words, in addition to an overall intercept, for the dose from each exposure source there was an estimated initial slope, an estimated downturn parameter, and an estimated overall slope. The model is

$p_{\theta}=\theta_{1}+\theta_{2}d\mathrm{e}^{-\theta_{3}d}+\theta_{4}d+\theta_{5}x\mathrm{e}^{-\theta_{6}x}+\theta_{7}x$ ,

where *d* is atomic-bomb radiation dose and *x* is medical diagnostic X-ray dose.

**Table S6. Constrained maximum likelihood parameter estimates for the basic four-parameter dose-response model (mother’s DS02R1 uterus dose, *d*) with full adjustment for in utero survivor’s estimated medical diagnostic X-ray dose (*x*)**

| **Parameter** | **Estimate** | **Standard error** |
| --- | --- | --- |
| DS02R1 and medical X-ray doses, 22 participants excluded (log-likelihood = −469.37) | | |
| *θ* _1_ — overall intercept (%) | 1.18 | 0.11 |
| *θ* _2_ — dose *d* initial slope (% / mGy) | 0.039 | 0.044 |
| *θ* _3_ — dose *d* downturn (mGy^−1^) | 0.037 | 0.022 |
| *θ* _4_ — dose *d* overall slope (% / Gy) | 0.372 | 0.370 |
| *θ* _5_ — dose *x* initial slope (% / mGy) | 0.000000016 | 0.00000013 |
| *θ* _6_ — dose *x* downturn (mGy^−1^) | 0.043 | Not estimable |
| *θ* _7_ — dose *x* overall slope (% / Gy) | 1.19 | 1.16 |
| DS02R1 dose only, 22 participants excluded ^a^ (log-likelihood = −469.99) | | |
| *θ* _1_ — intercept (%) | 1.26 | 0.084 |
| *θ* _2_ — dose *d* initial slope (% / mGy) | 0.036 | 0.041 |
| *θ* _3_ — dose *d* downturn (mGy^−1^) | 0.036 | 0.021 |
| *θ* _4_ — dose *d* overall slope (% / Gy) | 0.401 | 0.369 |
| DS02R1 dose only, all 330 participants ^b^ (log-likelihood = −503.18) | | |
| *θ* _1_ — intercept (%) | 1.26 | 0.081 |
| *θ* _2_ — dose *d* initial slope (% / mGy) | 0.037 | 0.053 |
| *θ* _3_ — dose *d* downturn (mGy^−1^) | 0.042 | 0.032 |
| *θ* _4_ — dose *d* overall slope (% / Gy) | 0.451 | 0.372 |
| ^a^ Log-likelihood values from fits of models with and without adjustment for medical X-ray dose cannot be directly compared because 22 individuals had unknown values of medical X-ray dose and hence were excluded from the fit adjusted for it; we therefore fit the model for DS02R1 dose only with those 22 individuals excluded  ^b^ Estimates for the model fit to DS02R1 doses only with data from all 330 participants are from Table 2 of the main manuscript | | |

With such adjustment for medical diagnostic X-ray dose, the parameter estimates for atomic-bomb radiation dose were virtually unchanged; they were also essentially unchanged when the model for atomic-bomb radiation dose only was fit with the 22 participants excluded who had unknown X-ray dose.

In addition to the above model where all parameters of the atomic-bomb radiation dose response were mimicked by the medical diagnostic X-ray dose, we fit a model with adjustment for X-ray dose using only a simple linear term:

$p_{\theta}=\theta_{1}+\theta_{2}d\mathrm{e}^{-\theta_{3}d}+\theta_{4}d+\theta_{5}x$ .

The parameter estimates obtained with this model are shown in the following table (Table S7).

Table S7. Constrained maximum likelihood parameter estimates for the basic four-parameter dose-response model (mother’s DS02R1 uterus dose, *d*) with linear adjustment for in utero survivor’s estimated medical diagnostic X-ray dose (*x*)

| **Parameter** | **Estimate** | **Standard error** |
| --- | --- | --- |
| DS02R1 and medical X-ray doses, 22 participants excluded (log-likelihood = −469.37) | | |
| *θ* _1_ — overall intercept (%) | 1.18 | 0.11 |
| *θ* _2_ — dose *d* initial slope (% / mGy) | 0.035 | 0.038 |
| *θ* _3_ — dose *d* downturn (mGy^−1^) | 0.035 | 0.020 |
| *θ* _4_ — dose *d* overall slope (% / Gy) | 0.372 | 0.369 |
| *θ* _5_ — dose *x* overall slope (% / Gy) | 1.21 | 1.16 |
|  | | |

Again there was virtually no change in the atomic-bomb dose-response parameter estimates with adjustment for estimated cumulative medical diagnostic X-ray dose, and the log-likelihood is the same as that with fuller adjustment for X-ray dose.

This is not a true mediation analysis, in part because there is no model for the causal effect of atomic-bomb dose on subsequent medical diagnostic X-ray dose. However, it seems unlikely that estimated medical diagnostic X-ray dose acts as a strong mediator, given that the atomic-bomb radiation dose-response coefficient parameter estimates were essentially unchanged with adjustment for medical diagnostic X-ray dose. Furthermore, despite a large overall linear coefficient, there is no clear evidence of an association between TF% and medical diagnostic X-ray dose in the above tables (Tables S6 and S7), nor was there evidence of such an association in nonparametric smooths (not shown). This lack of evidence of a dose response for medical diagnostic X-rays is perhaps not inconceivable, for several reasons: the total doses were rather low; the exposures would have been localized, rather than whole-body; there could be measurement error in the X-ray dose estimates; and the sample size is small. In conclusion, it seems likely that the estimated X-ray doses do not mediate the TF% dose response for A-bomb radiation dose in a practically significant sense, but rather contribute uniformly to TF% across the full A-bomb dose range. This conclusion is consistent with the fact that adjustment for X-ray doses in the above tables led to a reduction in the overall TF% intercept (from 1.26% without adjustment to 1.18% with adjustment).

These conclusions should be considered preliminary, however, until more reliable medical exposure dosimetry becomes available and an appropriate method of mediation analysis can be formulated.
